# Effect of Etomidate vs Propofol for Total Intravenous Anesthesia on Major Postoperative Complications in Older Patients: A Randomized Clinical Trial

**DOI:** 10.1001/jamasurg.2022.3338

**Published:** 2022-08-10

**Authors:** Zhihong Lu, Hong Zheng, Zhijun Chen, Shiyuan Xu, Shibiao Chen, Weidong Mi, Tianlong Wang, Xiaoqing Chai, Qulian Guo, Hai Zhou, Yonghao Yu, Xiaochun Zheng, Jiaqiang Zhang, Yanqiu Ai, Buwei Yu, Hongguang Bao, Hui Zheng, Wenqi Huang, Anshi Wu, Xiaoming Deng, Hong Ma, Weiqing Ma, Liyuan Tao, Xue Yang, Junbao Zhang, Tingting Liu, Hai-ping Ma, Wei Liang, Xiang Wang, Yang Zhang, Wei Du, Ting Ma, Yanhu Xie, Yongqiu Xie, Na Li, Yong Yang, Ting Zheng, Chunyan Zhang, Yanling Zhao, Rong Dong, Chen Zhang, Guohua Zhang, Kuanzhi Liu, Yan Wu, Xiaohua Fan, Wenfei Tan, Na Li, Hailong Dong, Lize Xiong

**Affiliations:** 1 Department of Anesthesiology and Perioperative Medicine, Xijing Hospital, Fourth Military Medical University, Xi’an, Shaanxi, China; 2 Department of Anesthesiology, The First Affiliated Hospital of Xinjiang Medical University, Urumuqi, Xinjiang, China; 3 Department of Anesthesiology, Affiliated Hospital of Guilin Medical University, Guilin, Guangxi, China; 4 Department of Anesthesiology, ZhuJiang Hospital of Southern Medical University, Guangzhou, Guangdong, China; 5 Department of Anesthesiology, The First Affiliated Hospital of Nanchang University, Nanchang, Jiangxi, China; 6 Department of Anesthesiology, Chinese PLA General Hospital, Peking, China; 7 Department of Anesthesiology, Xuanwu Hospital, Capital Medical University, Peking, China; 8 Department of Anesthesiology, Anhui Provincial Hospital, University of Science and Technology of China, Hefei, Anhui, China; 9 Department of Anesthesiology, Xiangya Hospital of Central South University, Changsha, Hunan, China; 10 Department of Anesthesiology, Xuzhou Central Hospital, Southeast University, Xuzhou, Jiangsu, China; 11 Department of Anesthesiology, Tianjin Medical University General Hospital, Tianjin, China; 12 Department of Anesthesiology, Fujian Provincial Hospital, Fuzhou, Fujian, China; 13 Department of Anesthesiology and Perioperative Medicine, People’s Hospital of Zhengzhou University, Zhengzhou, Henan, China; 14 Department of Anesthesiology, The First Affiliated Hospital of Zhengzhou University, Zhengzhou, Henan, China; 15 Department of Anesthesiology, Ruijin Hospital, School of Medicine, Shanghai Jiao Tong University, Shanghai, China; 16 Department of Anesthesiology, Nanjing First Hospital, Nanjing Medical University, Nanjing, Jiangsu, China; 17 Department of Anesthesiology, Cancer Hospital, Chinese Academy of Medical Sciences, Peking, China; 18 Department of Anesthesiology, The First Affiliated Hospital, Sun Yat-sen University, Guangzhou, Guangdong, China; 19 Department of Anesthesiology, Beijing Chaoyang Hospital, Capital Medical University, Peking, China; 20 Department of Anesthesiology, Changhai Hospital, Navy Medical University, Shanghai, China; 21 Department of Anesthesiology, The First Affiliated Hospital of China Medical University, Shenyang, Liaoning, China; 22 Department of Anesthesiology, Kunming General Hospital of Chengdu Military Region, Kunming, Yunnan, China; 23 Research Center of Clinical Epidemiology, Peking University Third Hospital, Peking, China; 24 Translational Research Institute of Brain and Brain-Like Intelligence and Department of Anesthesiology and Perioperative Medicine, Shanghai Fourth People’s Hospital Affiliated to Tongji University School of Medicine, Shanghai, China

## Abstract

**Importance:**

Older patients may benefit from the hemodynamic stability of etomidate for general anesthesia. However, it remains uncertain whether the potential for adrenocortical suppression with etomidate may increase morbidity.

**Objective:**

To test the primary hypothesis that etomidate vs propofol for anesthesia does not increase in-hospital morbidity after abdominal surgery in older patients.

**Design, Setting, and Participants:**

This multicenter, parallel-group, noninferiority randomized clinical trial (Etomidate vs Propofol for In-hospital Complications [EPIC]) was conducted between August 15, 2017, and November 20, 2020, at 22 tertiary hospitals in China. Participants were aged 65 to 80 years and were scheduled for elective abdominal surgery. Patients and outcome assessors were blinded to group allocation. Data analysis followed a modified intention-to-treat principle.

**Interventions:**

Patients were randomized 1:1 to receive either etomidate or propofol for general anesthesia by target-controlled infusion.

**Main Outcomes and Measures:**

Primary outcome was a composite of major in-hospital postoperative complications (with a noninferiority margin of 3%). Secondary outcomes included intraoperative hemodynamic measurements; postoperative adrenocortical hormone levels; self-reported postoperative pain, nausea, and vomiting; and mortality at postoperative months 6 and 12.

**Results:**

A total of 1944 participants were randomized, of whom 1917 (98.6%) completed the trial. Patients were randomized to the etomidate group (n = 967; mean [SD] age, 70.3 [4.0] years; 578 men [59.8%]) or propofol group (n = 950; mean [SD] age, 70.6 [4.2] years; 533 men [56.1%]). The primary end point occurred in 90 of 967 patients (9.3%) in the etomidate group and 83 of 950 patients (8.7%) in the propofol group, which met the noninferiority criterion (risk difference [RD], 0.6%; 95% CI, –1.6% to 2.7%; *P* = .66). In the etomidate group, mean (SD) cortisol levels were lower at the end of surgery (4.8 [2.7] μg/dL vs 6.1 [3.4] μg/dL; *P* < .001), and mean (SD) aldosterone levels were lower at the end of surgery (0.13 [0.05] ng/dL vs 0.15 [0.07] ng/dL; *P* = .02) and on postoperative day 1 (0.14 [0.04] ng/dL vs 0.16 [0.06] ng/dL; *P* = .001) compared with the propofol group. No difference in mortality was observed between the etomidate and propofol groups at postoperative month 6 (2.2% vs 3.0%; RD, –0.8%; 95% CI, –2.2% to 0.7%) and 12 (3.3% vs 3.9%; RD, –0.6%; 95% CI, –2.3% to 1.0%). More patients had pneumonia in the etomidate group than in the propofol group (2.0% vs 0.3%; RD, 1.7%; 95% CI, 0.7% to 2.8%; *P* = .001). Results were consistent in the per-protocol population.

**Conclusions and Relevance:**

Results of this trial showed that, compared with propofol, etomidate anesthesia did not increase overall major in-hospital morbidity after abdominal surgery in older patients, although it induced transient adrenocortical suppression.

**Trial Registration:**

ClinicalTrials.gov Identifier: NCT02910206

## Key Points

**Question:**

Does etomidate compared with propofol provide a noninferior effect on in-hospital morbidity when used for induction and maintenance of general anesthesia in older patients undergoing abdominal surgery?

**Findings:**

In this randomized clinical trial involving 1944 older patients who underwent elective abdominal surgery, the rate of major in-hospital complications was noninferior between patients who received etomidate and those who received propofol for general anesthesia (9.3% vs 8.7%).

**Meaning:**

Findings of this trial indicate that etomidate anesthesia does not increase postoperative morbidity in older patients compared with propofol.

## Teaser

This randomized clinical trial compares the in-hospital morbidity, clinical outcomes, and mortality associated with use of etomidate vs propofol during abdominal surgery in older patients in China.

## Introduction

Major postoperative complications contribute to adverse outcomes and high resource use in older patients.^1,2^ Etomidate and propofol are widely used general anesthetics. Although etomidate may be advantageous for induction of anesthesia in patients at high risk for perioperative morbidity and mortality because of its hemodynamic stability, concerns regarding relative adrenal insufficiency and its potential impact on outcomes may lead many anesthesiologists and anesthesia practitioners to instead favor propofol in this setting. However, there is limited evidence regarding outcomes of etomidate in high-risk patients, especially when used for anesthesia maintenance.

In patients with trauma,^3^ patients undergoing cardiac surgery,^4^ and even patients with critical illness,^5,6^ single-dose etomidate for induction of anesthesia showed no difference in mortality compared with other agents, whereas the influence of etomidate on morbidity, such as hospital-acquired pneumonia, remains controversial.^4,7,8,9^ Furthermore, because most studies have focused on bolus administration of etomidate for anesthesia induction, there is a lack of data on the effects of continuous infusion of etomidate for anesthesia maintenance on hemodynamics, adrenocortical suppression, and clinical outcomes.

We conducted the randomized clinical trial EPIC (Etomidate vs Propofol for In-hospital Complications) to test the primary hypothesis that etomidate for induction and maintenance of anesthesia does not increase in-hospital morbidity after abdominal surgery in older patients compared with propofol. We also compared mortality rates with etomidate vs propofol at 6 and 12 months after surgery.

## Methods

### Study Design and Population

EPIC was a multicenter, parallel-group, noninferiority randomized clinical trial conducted at 22 tertiary hospitals in China. The study protocol and statistical analysis plan are provided in Supplement 1. The study design was approved by the institutional ethics committee at each hospital before enrollment. All participants provided written informed consent before study entry. Data analyses were performed at the Research Center of Clinical Epidemiology of the Peking University Third Hospital in Beijing, China. We followed the Consolidated Standards of Reporting Trials (CONSORT) reporting guideline.

From August 15, 2017, through November 20, 2019, patients were assessed for eligibility on the day before their surgery. Eligible patients were aged 65 to 80 years and were scheduled for elective abdominal surgery. Key exclusion criteria were American Society of Anesthesiologists (ASA) Physical Status Classification System class higher than 3; body mass index (calculated as weight in kilograms divided by height in meters squared) lower than 18.5 or higher than 29.9; anticipated surgery duration of less than 1 hour or more than 4 hours; cerebrovascular accident, myocardial infarction, or unstable angina within the previous 3 months; severe hepatic or kidney dysfunction; preoperative blood pressure of 180/110 mm Hg or higher; surgery within the previous 3 months; and participation in another study within the previous 30 days. The 12 months of follow-up ended on November 20, 2020.

### Procedures

**Figure 1.**
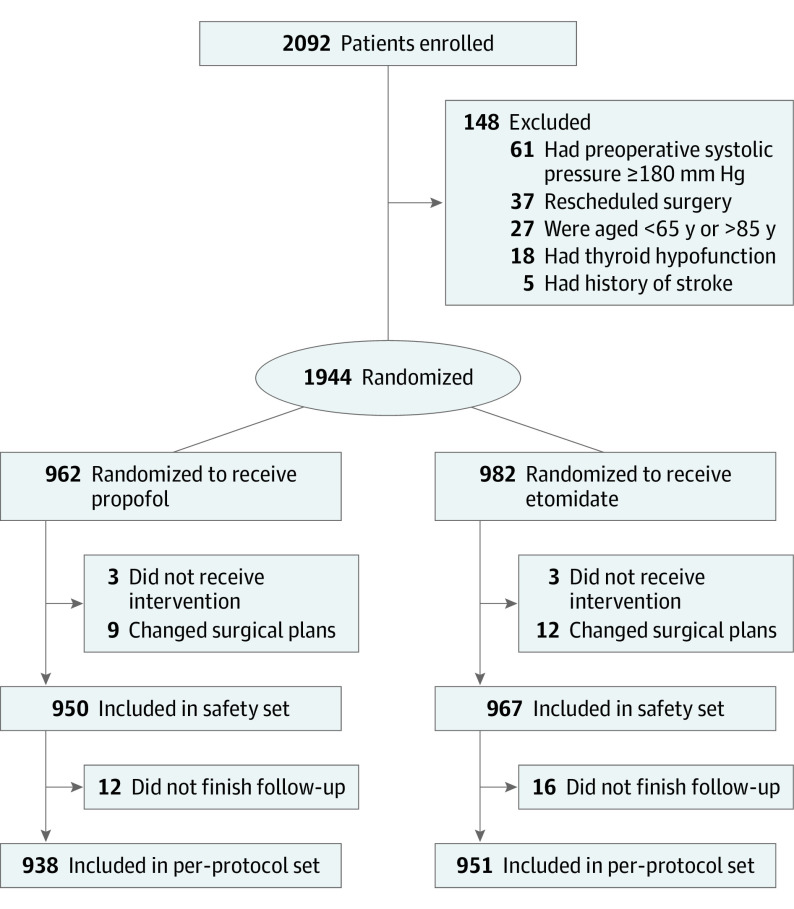
Patient Flowchart

Participants were randomized 1:1 to receive either etomidate or propofol for general anesthesia (Figure 1). Randomization was performed by investigators at each hospital using a central, web-based randomization system that concealed the allocation sequence. The random sequence was computer generated, had random block size, and was stratified by hospital. Investigators who performed the intervention were not blinded to group allocation; however, allocation was not revealed until the investigators accessed the web-based randomization system approximately 1 hour before surgery. Outcome assessors, other clinical staff (including surgeons, anesthesiologists who were not involved in etomidate or propofol administration, and hospital ward staff), the independent statistician, and patients were blinded.

Etomidate and propofol were administered by target-controlled infusion. For etomidate, the target plasma concentration was 0.5 to 0.8 μg/mL for induction and 0.2 to 0.4 μg/mL for maintenance of general anesthesia. For propofol, the target plasma concentration was 2 to 4 μg/mL for both induction and maintenance. Sufentanil and cisatracurium were also administered for general anesthesia in both intervention groups. Infusions were titrated to maintain a Narcotrend Index of 27 to 60 or a Bispectral Index of 40 to 60 during surgery. Hemodynamic stability during anesthesia and time to recovery from anesthesia were recorded. If required, nicardipine was administered for hypertension or norepinephrine for hypotension.

At the end of surgery, infusion of etomidate or propofol was discontinued. Patients were called by their name, and the time to response to verbal command was recorded. Sufentanil was used for patient-controlled analgesia. At 6 hours after surgery, postoperative nausea and vomiting (PONV) was scored using the World Health Organization scale, and patients were asked to report pain using a visual analog scale.

Patients were followed up every morning until discharge to identify any complications and PONV and visual analog scale pain scores for the preceding 24 hours. On the day after surgery, patients were asked to rate on a 1-to-10 scale their comfort (1 indicated very uncomfortable, and 10 indicated very comfortable) and satisfaction with anesthesia (1 indicated very unsatisfactory, and 10 indicated very satisfactory). At 5 centers, venous blood samples were collected at 7 am on the day of surgery, at the end of anesthesia, and at 7 am on postoperative days 1 and 3. Serum cortisol, aldosterone, and corticotropin (formerly adrenocorticotropic hormone) levels were measured by radioimmunoassay. Mortality was followed up by telephone contact with patients at postoperative months 6 and 12.

### End Points and Sample Size Calculation and Power

The primary end point was a composite of major in-hospital postoperative complications,^10^ as defined in the eMethods in Supplement 2, during the hospital stay. Secondary end points included time to response to verbal command and time to extubation; time to discharge from the postanesthesia care unit; patient self-reported pain as well as comfort and satisfaction with anesthesia; hypertension and hypotension incidence; PONV scores; serum concentrations of cortisol, aldosterone, and corticotropin at the end of anesthesia and at 7 am on postoperative days 1 and 3; and all-cause mortality at postoperative months 6 and 12.

The primary objective was to evaluate whether etomidate was noninferior to propofol in terms of the rate of major in-hospital complications after abdominal surgery in older patients. According to a preliminary study, the expected complication incidence was 7.5% in the etomidate group and 8.0% in the propofol group. The Δ was set at 3% based on expert advice. With α = .025 and β = 0.2, the number needed to demonstrate noninferiority was 917 patients per group (total sample size: 1834). Assuming a 5% ineligibility rate, 1930 patients were required to undergo screening.

### Statistical Analysis

Primary and secondary end points were analyzed according to a modified intention-to-treat principle in the safety set. The safety set comprised all patients who received the randomized intervention, underwent planned surgery, and continued to consent to follow-up. Mortality and hormone levels were analyzed among patients in the safety set who had available data. The multiple imputation method was used to account for missing data on mortality at postoperative months 6 and 12. Demographic characteristics were included in the imputed missing data model, and the number of multiple imputations was 5. Race and ethnicity data were not collected because all patients were of Han Chinese nationality. In addition, prespecified sensitivity analyses were conducted in the per protocol set, which included all patients in the safety set who completed follow-up, excluding those with protocol violations.

For the primary end point and categorical secondary end points, the absolute risk difference (RD) and associated 2-sided 95% CI were estimated using the Newcombe-Wilson score method.^11^ A generalized linear mixed model, including the hospital as a fixed effect, was used to control for possible differences between hospitals. Differences between intervention groups were assessed using the Fisher exact test or χ^2^ test. Survival was also analyzed using the Kaplan-Meier method and log-rank test. For continuous secondary end points, normally distributed data were presented as means with SDs and were compared between groups using the independent, unpaired, 2-tailed *t* test, whereas non–normally distributed variables were presented as medians with IQRs and were compared using the Mann-Whitney test. Mean (95% CI) between-group differences in the median were calculated using the bootstrap method with 1000 replications. Subgroup analyses were performed according to ASA class, duration of anesthesia, and duration of surgery. Hormone levels were analyzed by analysis of variance for repeated measurements. For secondary end points, subgroup and hormone level analyses are considered to be exploratory.

Statistical analyses were conducted using SPSS, version 18.0 (IBM), and R, version 3.4.0 (R Foundation for Statistical Computing). Statistical significance was defined as *P* < .05 with 2-sided testing, except for blood pressure comparisons for which the significance level was adjusted by Bonferroni correction.

## Results

A total of 1944 patients were randomized, of whom 1917 (98.6%) completed the trial (Figure 1). Baseline clinical characteristics were well balanced between the etodimate group (n = 967; mean [SD] age, 70.3 [4.0] years; 389 women [40.2%], 578 men [59.8%]) and propofol group (n = 950; mean [SD] age, 70.6 [4.2] years; 417 women [43.9%], 533 men [56.1%]) (Table 1).

**Table 1.** Patient Characteristics in the Safety Set^a^

Characteristic	Etomidate group (n = 967)	Propofol group (n = 950)
Age, mean (SD), y	70.3 (4.0)	70.6 (4.2)
Sex, No. (%)		
Female	389 (40.2)	417 (43.9)
Male	578 (59.8)	533 (56.1)
Height, mean (SD), cm	164.2 (7.4)	163.6 (8.0)
Weight, mean (SD), kg	62.7 (9.3)	62.6 (10.0)
BMI, mean (SD)	23.2 (2.8)	23.3 (2.8)
SBP, mean (SD), mm Hg	134.1 (15.7)	134.7 (15.2)
ASA Physical Status Classification System class, No. (%)		
1	23 (2.4)	21 (2.2)
2	719 (74.4)	684 (72.0)
3	225 (23.3)	245 (25.8)
History of comorbidity, No. (%)	26 (2.7)	25 (2.6)
History of anesthesia, No. (%)	9 (0.9)	11 (1.2)
History of allergy, No. (%)	2 (0.2)	2 (0.2)
Fluid input, median (IQR), mL	900 (600-1100)	900 (600-1200)
Surgery type, No. (%)		
Gastrointestinal	573 (59.3)	570 (60.0)
Hepatobiliary	394 (40.7)	380 (40.0)
Duration of surgery, median (IQR), h	2.1 (1.2-3.0)	2.0 (1.1-3.1)
Duration of anesthesia, median (IQR), h	2.4 (1.4-3.5)	2.5 (1.4-3.5)
Dose of sufentanil, median (IQR), μg	35.0 (30.0-40.0)	35.0 (30.0-40.0)
Dose of cisatracurium, median (IQR), mg	24.0 (17.2-31.0)	23.3 (17.0-30.8)

Abbreviations: ASA, American Society of Anesthesiologists; BMI, body mass index (calculated as weight in kilograms divided by height in meters squared); SBP, systolic blood pressure.

^a^ The safety set comprised all patients who received the randomized intervention, underwent planned surgery, and continued to consent to follow-up.

### Primary End Point

**Figure 2.**
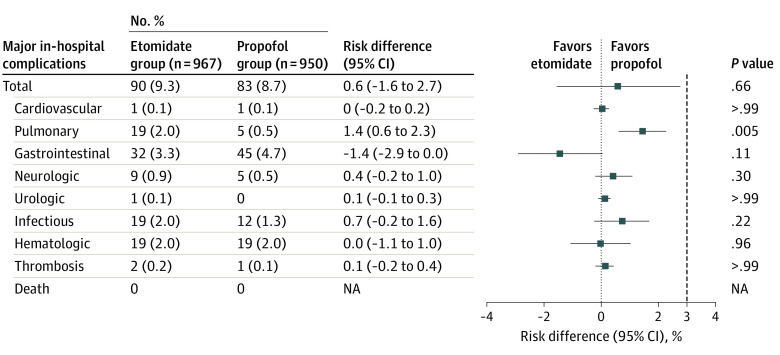
In-Hospital Complications for Etomidate and Propofol Groups NA indicates not applicable.

The primary end point of in-hospital major complications occurred in 90 of 967 participants (9.3%) in the etomidate group and 83 of 950 (8.7%) in the propofol group, which met the noninferiority criterion (RD, 0.6%; 95% CI, –1.6% to 2.7%; *P* = .66) (Figure 2). There were no significant between-group differences in individual complication types, except for pulmonary complications (2.0% [n = 19] vs 0.5% [n = 5]; RD, 1.4%; 95% CI, 0.6%-2.3%; *P* = .005) (Figure 2). Similar results were observed across subgroups, including ASA class, duration of anesthesia, and duration of surgery (eTable 1 in Supplement 2). For anesthesia duration of 2 hours or less, major in-hospital complications occurred in 6.9% of participants (34 of 492) in the etomidate group and 7.3% of participants (35 of 479) in the propofol group (RD, −0.4%; 95% CI, –3.1% to 2.3%; *P* = .81). For anesthesia duration of more than 2 hours, the corresponding rates were 11.2% of participants (62 of 554) in the etomidate group and 9.6% of participants (54 of 560) in the propofol group (RD, 1.5%; 95% CI, –1.5% to 4.6%; *P* = .40). After adjusting for hospital, the generalized linear mixed model analysis showed no significant difference in the primary end point between groups.

### Secondary End Points

Hemodynamic measurements are summarized in Table 2 and eFigure 1 in Supplement 2. Compared with patients in the propofol group, patients in the etomidate group had a higher rate of hypertension (3.8% vs 10.3%; *P* < .001) but a lower rate of hypotension (10.5% vs 2.4%; *P* < .001). Diastolic blood pressure was consistently higher in the etomidate group vs the propofol group at all postbaseline time points, except at 120 minutes after incision. Mean (SD) diastolic blood pressure was consistently higher in the etomidate group vs the propofol group at all postbaseline time points, except at 120 minutes after incision (69.4 [13.3] mm Hg vs 66.0 [27.1] mm Hg) (eFigure 1 in Supplement 2).

**Table 2.** Secondary End Points in the Safety Set

Outcome	Etomidate group (n = 967)	Propofol group (n = 950)	*P* value	Difference (95% CI)
During surgery				
Hypertension, No. (%)	100 (10.3)	36 (3.8)	<.001	6.5% (4.6% to 8.4%)
Hypotension, No. (%)	23 (2.4)	100 (10.5)	<.001	–8.1% (–10.0% to –6.3%)
During emergence				
Time to response to verbal command, median (IQR), min	10.8 (4.3 to 20.7)	11.9 (4.9 to 23.1)	.16	–1.1 (–1.6 to 0.2)
Duration of PACU stay, median (IQR), min	49.2 (32.1 to 69.9)	50.8 (33.7 to 69.9)	.15	–1.6 (–4.0 to 0.5)
During recovery				
PONV score ≥3, No. (%)				
Postoperative hour 6	131 (13.5)	131 (13.8)	.93	–0.3% (–2.8% to 2.3%)
Postoperative day 1	61 (6.3)	64 (6.7)	.73	–0.4% (–2.3% to 1.4%)
Postoperative day 3	7 (0.7)	12 (1.3)	.23	–0.5% (–1.3% to 0.2%)
Pain score, median (IQR)				
Postoperative hour 6	2 (2 to 3)	2 (1 to 3)	.05	0
Postoperative day 1	2 (1 to 3)	2 (1 to 3)	.81	0
Postoperative day 3	1 (0 to 2)	1 (0 to 2)	.36	0
Patient satisfaction with anesthesia rating, median (IQR)				
Postoperative day 1	8 (8 to 9)	8 (7 to 9)	.25	0
Patient comfort rating, median (IQR)				
Postoperative day 1	8 (7 to 9)	8 (7 to 9)	.008	0
Mortality, No. (%)^a^				
Postoperative month 6	15 (2.2)	20 (3.0)	.38	–0.8% (–2.2% to 0.7%)
Postoperative month 12	22 (3.3)	26 (3.9)	.53	–0.6% (–2.3% to 1.0%)

Abbreviations: PACU, postanesthesia care unit; PONV, postoperative nausea and vomiting.

^a^ Long-term survival data were available for 672 patients in the etomidate group and 664 patients in the propofol group.

Median (IQR) time to response to verbal command was shorter in the etomidate group compared with the propofol group (10.8 [4.3-20.7] minutes vs 11.9 [4.9-23.1] minutes; *P* = .16). Median (IQR) duration of postanesthesia care unit stay did not differ significantly between the 2 groups (49.2 [32.1-69.9] minutes vs 50.8 [33.7-69.9] minutes; *P* = .15) (Table 2). There were no significant between-group differences in the numbers of patients with PONV scores of 3 or higher or in pain scores at 6 hours, 1 day, or 3 days after surgery. On postoperative day 1, there was a significant difference between groups in median (IQR) patient comfort ratings (8 [7-9] vs 8 [7-9]; *P* = .008) but not in median (IQR) patient satisfaction with anesthesia ratings (Table 2; eTable 2 in Supplement 2).

**Figure 3.**
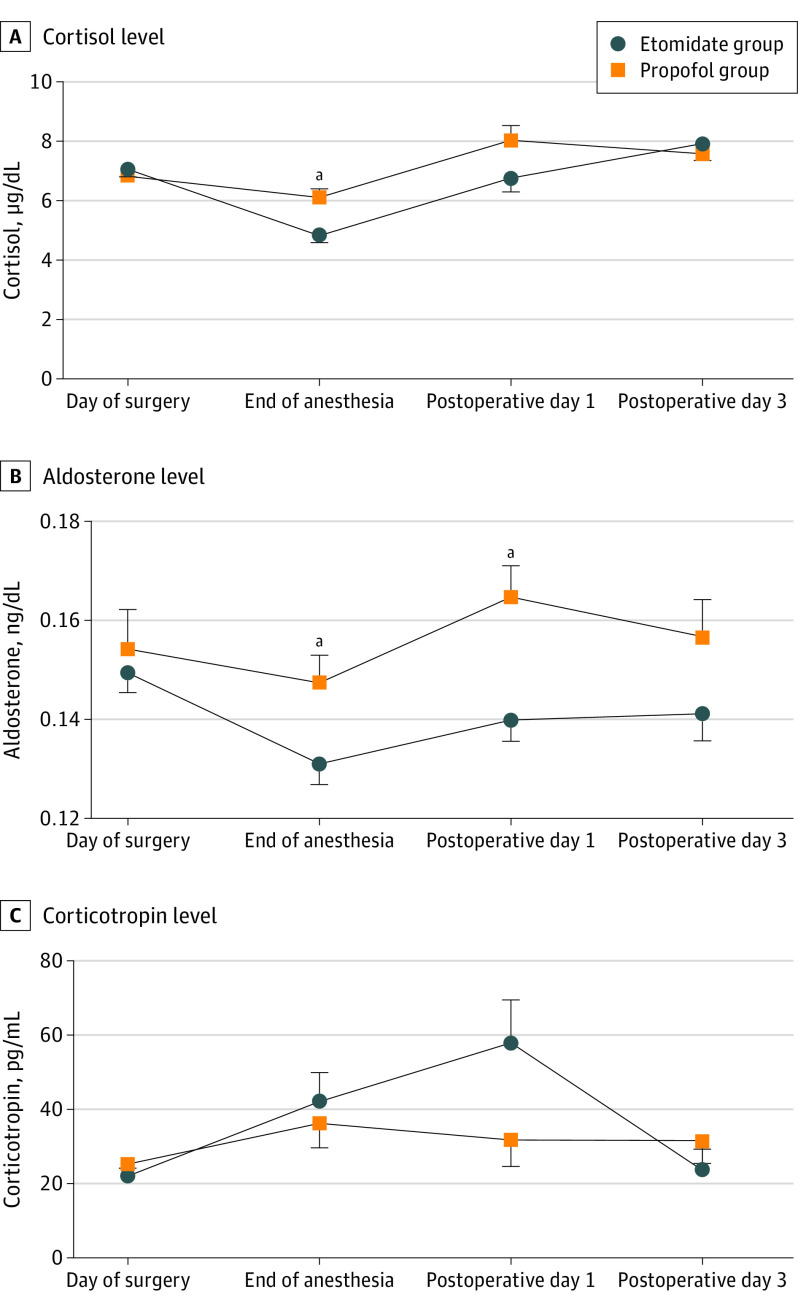
Mean Serum Concentrations of Cortisol, Aldosterone, and Corticotropin of Patients With Available Data in the Safety Set Error bars represent the SE of the mean. To convert cortisol to nanomoles per liter, multiply by 27.588; aldosterone to picomoles per liter, multiply by 27.74; and corticotropin to picomoles per liter, multiply by 0.22. ^a^Significant difference between groups.

Among patients with available blood samples (etomidate, n = 147; propofol, n = 149), the cortisol, aldosterone and corticotropin levels were similar between groups at baseline. Mean (SD) cortisol levels were significantly lower in the etomidate group than in the propofol group at the end of the surgery (4.8 [2.7] μg/dL vs 6.1 [3.4] μg/dL; *P* < .001); to convert to nanomoles per liter, multiply by 27.588. Mean (SD) aldosterone levels were lower in the etomidate vs the propofol group at the end of the surgery (0.13 [0.05] ng/dL vs 0.15 [0.07] ng/dL; *P* = .02) and on postoperative day 1 (0.14 [0.04] ng/dL vs 0.16 [0.06] ng/dL; *P* = .001); to convert to picomoles per liter, multiply by 27.74. Corticotropin levels did not differ significantly between the groups at any time point. Mean (SD) cortisol levels (4.8 [2.7] μg/dL vs 7.1 [3.1] μg/dL; *P* < .001) and aldosterone levels (0.13 [0.05] ng/dL vs 0.15 [0.05] ng/dL; *P* < .001) in the etomidate group were significantly lower at the end of surgery than before surgery and returned to baseline levels by 7 am on postoperative day 1 (6.8 [4.6] μg/dL and 0.14 [0.04] ng/dL) (Figure 3). The analysis of variance for repeated measurements found no differences between the groups in cortisol, aldosterone, or corticotropin levels. In the etomidate group, compared with baseline, cortisol concentration was significantly higher on postoperative day 3 (7.9 [5.1] μg/dL vs 7.1 [3.1] μg/dL; *P* = .03), and corticotropin concentration was significantly higher on postoperative day 1 (57.9 [115.0] vs 22.1 [25.8] pg/mL; *P* = .002); to convert corticotropin to picomoles per liter, multiply by 0.22. In the propofol group, compared with baseline, cortisol levels were significantly lower at the end of surgery (6.1 [3.4] μg/dL vs 6.8 [3.1] μg/dL; *P* = .04) but higher on postoperative day 1 (8.0 [5.1] μg/dL; *P* = .007) and day 3 (7.6 [4.3] μg/dL; *P* = .009); aldosterone levels were higher on postoperative day 1 (0.16 [0.06] ng/dL vs 0.15 [0.10] ng/dL; *P* = .02); and corticotropin levels did not change significantly (eTable 3 in Supplement 2).

Survival data at postoperative months 6 and 12 were available for 672 of 967 patients (69.5%) in the etomidate group and 664 of 950 patients (69.9%) in the propofol group in the safety set; the remaining patients were lost to follow-up. Mortality rates were similar between the etomidate and propofol groups at postoperative month 6 (2.2% vs 3.0%; RD, –0.8%; 95% CI, –2.2% to 0.7%; *P* = .38) and month 12 (3.3% vs 3.9%; RD, –0.6%; 95% CI, –2.3% to 1.0%; *P* = .53) (Table 2). There was no significant difference between groups in the Kaplan-Meier survival curves (eFigure 2 in Supplement 2). Sensitivity analysis after multiple imputation also found no statistically significant difference in mortality rates.

### Sensitivity Analysis

Compared with the main analyses in the safety set, sensitivity analyses in the per protocol set yielded consistent findings for both the primary outcome (major in-hospital complication rates: 9.4% in the etomidate group vs 8.8% in the propofol group; RD, 0.5%; 95% CI, –1.7% to 2.7%; *P* = .70) and secondary outcomes (eTable 4 in Supplement 2). Analysis of individual complications showed more incidence of pneumonia in the etomidate vs propofol group (2.0% vs 0.3%; RD, 1.7%; 95% CI, 0.7% to 2.8%; *P* = .001) (eTable 5 in Supplement 2).

## Discussion

In the EPIC trial, we found the noninferiority of etomidate compared with propofol in induction and maintenance of anesthesia for the primary outcome of major in-hospital complications in older patients who underwent abdominal surgery. To our knowledge, this randomized clinical trial is the first to examine etomidate general anesthesia in older patients and is the largest study to assess the effect of etomidate infusion on adrenal suppression and postoperative complications in this population.

Analysis of complication subtypes suggested an increase in pulmonary complications, specifically pneumonia, with etomidate vs propofol. This finding is consistent with previous reports that single-dose etomidate is associated with a higher incidence of hospital-acquired pneumonia after cardiac surgery^7^ and that etomidate is an independent risk factor for hospital-acquired pneumonia in patients with trauma.^9^ However, another retrospective study in patients with critical illness reported no association between single-dose etomidate and pneumonia.^8^ Nonetheless, the collective evidence supports cautious use of etomidate infusion in patients at risk for pulmonary complications. Given the relevance of pulmonary complications for mortality in older patients after major surgery, this issue requires further study.

Etomidate anesthesia (vs propofol) resulted in less hypotension but more hypertension during surgery. Improved hemodynamic stability observed with etomidate vs propofol has been reported in various studies, especially in patients with a high risk of mortality, including those with hemorrhagic shock^12^ or sepsis.^13^ Although hemorrhagic shock leads to substantially higher concentrations and increased effect of most anesthetic agents, including propofol, it does not affect the pharmacokinetics of etomidate.^12^ In this trial, higher blood pressure was observed with etomidate not only during induction but also throughout anesthesia. A systematic review of the association between intraoperative hypotension and adverse postoperative outcomes in noncardiac surgery suggests that organ injury might increase progressively as mean arterial pressure decreases below 80 mm Hg for 10 minutes or more.^14^ However, the influence of hypertension on postoperative morbidity is unclear.

At the end of surgery, cortisol and aldosterone levels decreased in the etomidate group, but the aldosterone level did not change in the propofol group. The lower cortisol and aldosterone levels in the etomidate group are consistent with findings of previous studies of single-dose etomidate.^15^ A subanesthetic dose of etomidate (3- to 5-mg loading dose followed by 0.03-0.10 mg/kg/h continuous infusion) has been used to control hypercortisolism in Cushing syndrome.^16,17^ Results of this trial indicated that total intravenous anesthesia with etomidate for less than 4 hours is not inferior to propofol with respect to postoperative outcomes. Furthermore, total intravenous anesthesia with etomidate may reduce the dose needed for induction and reduce the incidence of myoclonus, making it a safer choice for etomidate anesthesia.^18^ Changes in cortisol or aldosterone concentration in the etomidate group were not associated with changes in corticotropin concentration. This finding is consistent with a previous report on patients in intensive care units,^19^ which suggested that reductions in cortisol and aldosterone levels were not mediated centrally via corticotropin level but rather peripherally via the effects on the adrenal cortex.

Suppressed cortisol levels returned to baseline by postoperative day 3 after etomidate infusion. Cortisol recovery could be as fast as within 24 hours after a single dose of etomidate according to studies involving patients with heart failure,^20,21^ patients who underwent cardiac surgery,^22^ patients with morbid obesity,^23^ and otherwise healthy children who underwent urologic surgery.^24^ An analog of etomidate has been developed to retain the beneficial hemodynamic profile of etomidate without adrenocortical suppression.^25^ There is no evidence that corticosteroid supplementation after etomidate can improve outcomes, including 28-day mortality, ventilation days, or duration of intensive care unit stay.^26,27,28^

Mortality rates at postoperative months 6 and 12 were not significantly different between the etomidate and propofol groups. This finding is consistent with results of a previous study that showed no association between etomidate and in-hospital or 30-day mortality in patients with cardiac failure.^20^ Although a retrospective study of patients with ASA class 3 or 4 who underwent noncardiac surgery suggested an association between etomidate and increased risk of 30-day mortality, cardiovascular morbidity, and prolonged hospital stay, the analysis did not perform propensity matching for confounding variables or selection bias.^29^ A meta-analysis of 14 trials found increased mortality in patients with critical illness who received single-dose etomidate^15^; however, we observed no difference in the primary end point between etomidate and propofol in the ASA class 3 subgroup.

### Limitations

This study has several limitations. First, it is unclear whether the results can be generalized to surgery that lasts more than 4 hours or to populations outside of China. Second, the duration and frequency of hypotensive or hypertensive events were not analyzed. Third, although the primary composite end point was designed to provide a comprehensive picture of postoperative sequelae, treatment effects in opposite directions for the component events may have offset one another. Fourth, because analyses of individual complication types or other secondary end points may have been underpowered and were not adjusted for multiplicity, those findings should be regarded as exploratory. Fifth, although all primary end point events represent major postoperative complications, potential differences in clinical significance between event types were not reflected in the analysis, which weighted all events equally. Sixth, because the anesthetic dose was titrated according to target-controlled infusion, with different target concentrations for each agent, it would have been difficult to blind the investigators who performed the intervention to group allocation. However, blinding of the patients, surgeons, outcome assessors, and statisticians may have minimized observer bias.

## Conclusions

In this EPIC trial involving older patients who underwent abdominal surgery, total intravenous anesthesia with etomidate infusion did not increase overall major in-hospital morbidity compared with propofol anesthesia despite its transient adrenocortical suppression.

## Notes

Supplement 1.
Trial Protocol and Statistical Analysis Plan

Supplement 2. **eMethods.** Definition of Major Complications
eReference
**eTable 1.** Subgroup Analysis of the Effect of Etomidate vs Propofol on Major In-Hospital Complications (Safety Set) **eTable 2.** Mean (SD) Pain, Patient Satisfaction with Anesthesia and Patient Comfort Scores (Safety Set) **eTable 3.** ANOVA for Repeated Measurements of Serum Cortisol, Aldosterone and ACTH (Safety Set) **eTable 4.** Primary and Secondary End Points (Per Protocol Set) **eTable 5.** Effect of Etomidate vs Propofol on Individual Complications (Safety Set) **eFigure 1.** Blood Pressure, Heart Rate and Pulse Oximetry During Anesthesia (Safety Set) **eFigure 2.** Kaplan-Meier Curves of Survival After Surgery (Safety Set)

Supplement 3.
Data Sharing Statement

## References

[1] Gajdos C, Kile D, Hawn MT, Finlayson E, Henderson WG, Robinson TN. Advancing age and 30-day adverse outcomes after nonemergent general surgeries. J Am Geriatr Soc. 2013;61(9):1608-1614. doi:10.1111/jgs.12401 23927841 PMC4119758

[2] Gleason LJ, Schmitt EM, Kosar CM, . Effect of delirium and other major complications on outcomes after elective surgery in older adults. JAMA Surg. 2015;150(12):1134-1140. doi:10.1001/jamasurg.2015.2606 26352694 PMC4684425

[3] Gäßler M, Ruppert M, Lefering R, Bouillon B, Wafaisade A; TraumaRegister DGU. Pre-hospital emergent intubation in trauma patients: the influence of etomidate on mortality, morbidity and healthcare resource utilization. Scand J Trauma Resusc Emerg Med. 2019;27(1):61. doi:10.1186/s13049-019-0637-z 31174573 PMC6555933

[4] Yao YT, He LX, Fang NX, Ma J. Anesthetic induction with etomidate in cardiac surgical patients: a PRISMA-compliant systematic review and meta-analysis. J Cardiothorac Vasc Anesth. 2021;35(4):1073-1085. doi:10.1053/j.jvca.2020.11.068 33384231

[5] Albert SG, Sitaula S. Etomidate, adrenal insufficiency and mortality associated with severity of illness: a meta-analysis. J Intensive Care Med. 2021;36(10):1124-1129. doi:10.1177/0885066620957596 32912050

[6] Cagliani JA, Ruhemann A, Molmenti E, Smith C, Coppa G, Barrera R. Association between etomidate use for rapid sequence intubation and adrenal insufficiency in sepsis. Cureus. 2021;13(2):e13445. doi:10.7759/cureus.13445 33767929 PMC7982295

[7] Weiss B, Schiefenhövel F, Grunow JJ, . Infectious complications after etomidate vs. propofol for induction of general anesthesia in cardiac surgery—results of a retrospective, before–after study. J Clin Med. 2021;10(13):2908. doi:10.3390/jcm10132908 34209919 PMC8269440

[8] Hammond DA, Vines CE, McPhee AL, . Effect of etomidate on pneumonia development in critically ill, nontrauma patients. J Intensive Care Med. 2019;34(1):34-39. doi:10.1177/0885066616686052 28027685

[9] Asehnoune K, Mahe PJ, Seguin P, . Etomidate increases susceptibility to pneumonia in trauma patients. Intensive Care Med. 2012;38(10):1673-1682. doi:10.1007/s00134-012-2619-8 22777514

[10] Futier E, Lefrant JY, Guinot PG, ; INPRESS Study Group. Effect of individualized vs standard blood pressure management strategies on postoperative organ dysfunction among high-risk patients undergoing major surgery: a randomized clinical trial. JAMA. 2017;318(14):1346-1357. doi:10.1001/jama.2017.14172 28973220 PMC5710560

[11] Newcombe RG. Interval estimation for the difference between independent proportions: comparison of eleven methods. Stat Med. 1998;17(8):873-890. doi:10.1002/(SICI)1097-0258(19980430)17:8<873::AID-SIM779>3.0.CO;2-I 9595617

[12] Egan ED, Johnson KB. The influence of hemorrhagic shock on the disposition and effects of intravenous anesthetics: a narrative review. Anesth Analg. 2020;130(5):1320-1330. doi:10.1213/ANE.0000000000004654 32149755

[13] Gu WJ, Wang F, Tang L, Liu JC. Single-dose etomidate does not increase mortality in patients with sepsis: a systematic review and meta-analysis of randomized controlled trials and observational studies. Chest. 2015;147(2):335-346. doi:10.1378/chest.14-1012 25255427

[14] Wesselink EM, Kappen TH, Torn HM, Slooter AJC, van Klei WA. Intraoperative hypotension and the risk of postoperative adverse outcomes: a systematic review. Br J Anaesth. 2018;121(4):706-721. doi:10.1016/j.bja.2018.04.036 30236233

[15] Albert SG, Ariyan S, Rather A. The effect of etomidate on adrenal function in critical illness: a systematic review. Intensive Care Med. 2011;37(6):901-910. doi:10.1007/s00134-011-2160-1 21373823

[16] Nieman LK, Biller BM, Findling JW, ; Endocrine Society. Treatment of Cushing’s syndrome: an Endocrine Society clinical practice guideline. J Clin Endocrinol Metab. 2015;100(8):2807-2831. doi:10.1210/jc.2015-1818 26222757 PMC4525003

[17] Preda VA, Sen J, Karavitaki N, Grossman AB. Etomidate in the management of hypercortisolaemia in Cushing’s syndrome: a review. Eur J Endocrinol. 2012;167(2):137-143. doi:10.1530/EJE-12-0274 22577107

[18] Prakash MVSS, Gnanasekar R, Sakthirajan P, Adole PS. A comparative study of two infusion doses of etomidate for induction vs standard induction dose of etomidate. Eur J Clin Pharmacol. 2019;75(7):889-894. doi:10.1007/s00228-019-02681-6 31037454

[19] Peeters B, Güiza F, Boonen E, Meersseman P, Langouche L, Van den Berghe G. Drug-induced HPA axis alterations during acute critical illness: a multivariable association study. Clin Endocrinol (Oxf). 2017;86(1):26-36. doi:10.1111/cen.13155 27422812

[20] Chung M, Santer P, Raub D, . Use of etomidate in patients with heart failure undergoing noncardiac surgery. Br J Anaesth. 2020;125(6):943-952. doi:10.1016/j.bja.2020.06.059 32807381 PMC7729846

[21] Basciani RM, Rindlisbacher A, Begert E, . Anaesthetic induction with etomidate in cardiac surgery: a randomised controlled trial. Eur J Anaesthesiol. 2016;33(6):417-424. doi:10.1097/EJA.0000000000000434 26914224

[22] Komatsu R, Makarova N, You J, . Etomidate and the risk of complications after cardiac surgery: a retrospective cohort analysis. J Cardiothorac Vasc Anesth. 2016;30(6):1516-1522. doi:10.1053/j.jvca.2016.04.022 27554237

[23] Możański M, Tomaszewski D, Rybicki Z, Bejm J, Bałkota M. Etomidate, but not thiopental, decreases serum cortisol concentration in morbidly obese patients. A randomized controlled trial. Anaesthesiol Intensive Ther. 2016;48(1):7-12. doi:10.5603/AIT.2016.0002 26966106

[24] Du Y, Chen YJ, He B, Wang YW. The effects of single-dose etomidate versus propofol on cortisol levels in pediatric patients undergoing urologic surgery: a randomized controlled trial. Anesth Analg. 2015;121(6):1580-1585. doi:10.1213/ANE.0000000000000981 26496368

[25] Valk BI, Absalom AR, Meyer P, . Safety and clinical effect of i.v. infusion of cyclopropyl-methoxycarbonyl etomidate (ABP-700), a soft analogue of etomidate, in healthy subjects. Br J Anaesth. 2018;120(6):1401-1411. doi:10.1016/j.bja.2018.01.038 29793605

[26] Cuthbertson BH, Sprung CL, Annane D, . The effects of etomidate on adrenal responsiveness and mortality in patients with septic shock. Intensive Care Med. 2009;35(11):1868-1876. doi:10.1007/s00134-009-1603-4 19652948

[27] Payen JF, Dupuis C, Trouve-Buisson T, . Corticosteroid after etomidate in critically ill patients: a randomized controlled trial. Crit Care Med. 2012;40(1):29-35. doi:10.1097/CCM.0b013e31822d7938 21926601

[28] van den Heuvel I, Wurmb TE, Böttiger BW, Bernhard M. Pros and cons of etomidate—more discussion than evidence? Curr Opin Anaesthesiol. 2013;26(4):404-408. doi:10.1097/ACO.0b013e328362a84c 23743556

[29] Komatsu R, You J, Mascha EJ, Sessler DI, Kasuya Y, Turan A. Anesthetic induction with etomidate, rather than propofol, is associated with increased 30-day mortality and cardiovascular morbidity after noncardiac surgery. Anesth Analg. 2013;117(6):1329-1337. doi:10.1213/ANE.0b013e318299a516 24257383

