# Supplementary material for: Effect of Etomidate vs Propofol for Total Intravenous Anesthesia on Major Postoperative Complications in Older Patients: A Randomized Clinical Trial
Source: JAMA Surg. 2022 Aug 10;157(10):888–95. doi: 10.1001/jamasurg.2022.3338 (PMC9366659; doi:10.1001/jamasurg.2022.3338)
Supplement: Supplement 1. — Trial Protocol and Statistical Analysis Plan [file jamasurg-e223338-s001.pdf]

Supplement 1. Study Protocol

Effect of Etomidate and Propofol on postoperative complications in Elderly Patients (EPIC): A Multicenter, Randomized, Controlled Trial

Protocol

Central Site: Xijing Hospital, Fourth Military Medical University

Principle Investigator: Lize Xiong

Version number: V 5.0

Version date: 20170609

Table of Contents

1. **Background**
2. **Study objectives**
3. **Methods and Study design**
   - **3.1 Study Overview**
   - **3.2 Setting**
   - **3.3 Population**
   - **3.4 Intervention**
   - **3.5 Anesthesia protocol**
   - **3.6 Measurements**
   - **3.7 Outcomes**
   - **3.8 Data Collection**
   - **3.9 Statistical analysis**
   - **3.10 Sample size considerations**
4. **References**
5. **Appendix**

1. Background

A. Increasing incidence of postoperative complications and mortality in elderly patients

With the aging of the Chinese population and advancements in medical care, the number of surgeries performed on elderly patients (≥ 65 years) has increased, accounting for 35% of all surgeries.[1] Due to their reduced cardiopulmonary reserve, the rates of perioperative complications and mortality are higher in elderly patients.[2] In a study of 6,953 patients ≥ 18 years old who had undergone major surgeries, Florence et al. found that the incidence of postoperative complications increased linearly with age, with a 0.71% increase for every 1-year increase in age from 20 to 99 years old, and a significant positive correlation between age and mortality.[3] The incidence of postoperative cardiovascular complications also increases progressively with age.[3]

B. High incidence of postoperative cardiac complications and the serious impact on prognosis in elderly patients

Major cardiac complications occur in more than 1 million patients each year after non-cardiac surgeries, most of whom are elderly.[4] The incidence of cardiac events (e.g., myocardial infarction and cardiac death) in patients with cardiac disease undergoing non-cardiac surgery is 1% to 5%.[5,6] Additionally, patients who suffer from cardiac complications have a 6-fold increased risk of non-cardiac complications (e.g., wound infection, respiratory failure, deep vein thrombosis, cerebrovascular accident, renal failure, etc.) compared to those without cardiac complications.[7] One study showed that > 11% of patients who suffer perioperative myocardial infarctions die within 30 days of surgery,[8] and at least 10% of perioperative all-cause deaths are related to cardiac complications.[9] In addition, postoperative cardiovascular complications lead to a significant increase in length of stay, hospital costs, and risk of death.[10]

C. Intraoperative circulation management is closely associated with various postoperative complications

Preoperative cardiac risk assessments can be performed to prevent or manage the development of various postoperative complications, usually using a risk score or a risk stratification tool such as the Revised Cardiac Risk Index. However, the usefulness of these scores is limited because intraoperative events

associated with surgery can significantly alter cardiac risk.[4] After controlling for preoperative cardiac risk factors, one meta-analysis identified seven independent intraoperative risk factors for cardiac complications: duration of surgery, need for blood transfusion, tachycardia, hypertension, hypotension, hypothermia, and distal-site ischemic preconditioning.[11] Among these, hypertension, hypotension, and tachycardia all fall under the umbrella of circulatory management. Several studies have shown that hemodynamic stability is associated with perioperative cardiac complications and other complications such as cerebral infarction and acute kidney injury. For example, one study showed that a 40% reduction in mean arterial pressure (MAP) and a MAP < 50 mm Hg in intraoperative high-risk patients are both associated with the occurrence of cardiac events.[12] In non-cardiac surgery, even an intraoperative MAP < 55 mm Hg lasting only a short time can lead to acute kidney and myocardial injury.[13] In summary, intraoperative circulatory management is essential for reducing postoperative complications.

D. Optimize the selection of anesthetic to reduce postoperative complications

Some studies have shown that a prolonged increase or decrease of 20 mm Hg or more from baseline in intraoperative MAP can lead to a significant increase in fatal surgical complications such as myocardial infarction and ischemia. Anesthetic drugs are the most common reason for a decrease in blood pressure (BP) during surgery. After anesthesia, systemic vasodilation occurs and blood flow increases, while BP decreases. Therefore, the selection of drugs with low circulatory and respiratory effects may be one way to reduce the incidence of serious complications (i.e., cardiac complications). Propofol, a commonly-used intravenous anesthetic, inhibits myocardial contractility and peripheral pressure receptors and dilates peripheral vasculature, causing a decrease in BP. In contrast to propofol, etomidate has no effect on myocardial contractility, peripheral pressure receptors, or peripheral vasculature.[14,15] Therefore, we hypothesize that performing anesthesia with etomidate in elderly patients may reduce the occurrence of complications, especially those that severely affect prognosis, such as cardiac complications.

2. **Objective** The purpose of this multicenter trial is to study the effect of etomidate- and propofol-based intravenous anesthesia on in-hospital postoperative complications in elderly patients.

***Primary Aim*:** To assess whether etomidate based intravenous anesthesia is noninferior to propofol for the in-hospital major morbidity after surgery in elderly patients undergoing abdominal surgery.

Secondary Aims

A. To evaluate the effect of etomidate based intravenous anesthesia on postoperative adrenocortical function in elderly patients compared with propofol.

B. To evaluate the effect of etomidate based intravenous anesthesia on hemodynamic measurements compared with propofol.

C. To evaluate the effect of etomidate based intravenous anesthesia on emergence, pain and comfort after surgery compared with propofol.

D. To evaluate the effect of etomidate based intravenous anesthesia on mortality by 6m and 1y after surgery compared with propofol.

3. Methods and study design

3.1 Study Overview

The trial is a noninferiority, parallel group, randomized, multicenter clinical trial.

Ethical considerations

The study design has been approved by institutional ethics committees at each site. All potential patients need to provide written informed consent prior to study entry.

3.2 Setting

The trial will be conducted in 22 academic hospitals in China. The central site is Xijing Hospital of Fourth Military Medical University. The participating centers include:

The First Affiliated Hospital of Xinjiang Medical University

Affiliated Hospital of Guilin Medical University

ZhuJiang Hospital of Southern Medical University

The First Affiliated Hospital of Nanchang University

Chinese PLA General Hospital

Xuanwu Hospital Capital Medical University

Anhui Provincial Hospital, University of Science and Technology of China

Xiangya Hospital of Central South University

Xuzhou Central Hospital, Southeast University

Tianjin Medical University General Hospital

Fujian Provincial Hospital

People's Hospital of Zhengzhou University

The First Affiliated Hospital of Zhengzhou University

Ruijin Hospital Affiliated to Shanghai Jiao Tong University School of Medicine

Nanjing First Hospital, Nanjing Medical University

Cancer Hospital of Chinese Academy of Medical Sciences

The First Affiliated Hospital,Sun Yat-sen University

Beijing Chaoyang Hospital, Capital Medical University

Changhai hospital, Navy Medical University

The First Affiliated Hospital of China Medical University

Kunming General Hospital of Chengdu Military Region

3.3 Population

Inclusion criteria

1) Aged from 65 to 80 years

2) Patients undergoing laparoscopic or open abdominal surgery under general anesthesia

Exclusion criteria

1) ASA status >Ⅲ

2) Body mass index (BMI) less than 18.5 or higher than 29.9 kg/m^2 according to the 2013 US Guidelines for the Management of Overweight and Obesity in Adults [BMI = weight (kg)/height (m)^2]

3) Anticipated duration of surgery less than 1h or longer than 4 h

4) Patients with any cerebrovascular accident, such as stroke or transient ischemic attack, among others within the previous 3 months

5) Patients with severe abnormal liver function (evidenced by alanine aminotransferase, conjugated bilirubin, aspartate aminotransferase, alkaline phosphatase, or total bilirubin > 2 times the upper limit of the normal range) or renal function (creatinine clearance < 30 mL/min)

6) Diabetic patients with complications from diabetes (diabetic ketoacidosis, hyperosmolar coma, various infections, macroangiopathy, diabetic nephropathy, retinopathy, diabetic cardiomyopathy, diabetic neuropathy, diabetic foot, etc.)

7) Patients with unstable angina or myocardial infarction within the previous 3 months

8) Patients with a BP ≥ 180/110 mmHg at the preoperative visit

9) Patients with identified or suspected abuse or chronic use of narcotic sedative analgesics

10) Patients taking hormones or other immunosuppressive drugs for > 10 days of the previous 6 months or with a history of adrenocortical suppression or immune system disorders

11) Patients with low thyroid function

12) Patients with a history of asthma

13) Patients who underwent another operation in the previous 3 months

14) Patients with contraindications or allergies to the test drugs or other anesthetic drugs

15) Patients enrolled in other studies within the previous 30 days

Withdrawal criteria

1) Patients who either do not meet the inclusion criteria or meet the exclusion criteria

2) Duration of surgery > 4h or <1h; intraoperative mass hemorrhage (blood loss > 800 mL, external gastrointestinal hemorrhage); or unintended injure to vital organs such as the liver, kidneys, or pancreas

3) Other conditions that the researcher considers to be grounds for exclusion

4) Intention to withdraw of the patient

3.4 Intervention

Study groups and Randomization

Participants are randomized into two groups: the etomidate group and the propofol group. Local investigators randomly allocate participants after their enrolment using a secure, central web-based randomization system. The random sequence will be generated by a computer with a block size of four, stratified by centers.

Research drugs

Name	Manufacturer	Formulation
Etomidate	Jiangsu Enhwa Pharmaceutical Co.	10 mL:20 mg
Propofol	AstraZeneca Pharmaceuticals UK Ltd.	10 mg/mL

Infusion equipment Three-channel intravenous target-controlled infusion pump (TCI-III; Guangxi Willie Ark Technology Development Co., Ltd.)

Administration After the patients are randomized, the drugs will be administered according to the method described below and the randomization number for that patient will be affixed to the syringe.

Etomidate: the recommended starting effective site target concentration is 0.5–0.8 μg/mL for anesthesia induction and 0.2–0.4 μg/mL for anesthesia maintenance; the researchers adjust the target concentration in a stepwise manner according to the patient’s condition

Propofol: the recommended starting effective site target concentration is 2–4 μg/mL for both anesthesia induction and maintenance; the researchers adjust the target concentration in a stepwise manner according to the patient’s condition

3.5 Anesthesia protocol

Pre-anesthesia management

Upper extremity non-invasive arterial pressure is measured during the preoperative visit, and invasive arterial BP monitoring is performed on the same, with heart rate (HR) and BP obtained from invasive arterial monitoring.The patient is admitted with upper extremity intravenous access, hydrated according to the fluid management method described below, and vital signs are monitored. Monitoring includes invasive arterial BP, HR, pulse oximetry (SpO_2), electrocardiogram, Narcotrend or BIS to monitor depth of anesthesia.

Fluid management

A goal-directed fluid management strategy is used. Pulse pressure variation (PPV) and/or stroke volume variation (SVV) are monitored. Lactated Ringer's solution is initiated at 5–7 mL/kg before and during induction. The recommended rehydration rate is 1–2 mL/kg/h during maintenance of laparoscopic surgery, and a moderate increase is acceptable for open surgery to maintain PPV or SVV < 13%. The total amount of rehydration fluids before intubation and during maintenance are recorded.

Body temperature management

Real-time intraoperative temperature monitoring is implemented and the central body temperature is maintained at a minimum of 36°C by means of an insulating blanket. An inflatable heater is routinely used to maintain body temperature after the patient is admitted to the post-anesthesia care unit (PACU) after surgery.

Anesthesia induction

Sufentanil of 0.5–0.8 μg/kg, cisatracurium of 0.15 mg/kg, and the TCI of propofol or etomidate at a target concentration described previously will be given for anesthesia induction. When the patient shows loss of consciousness and muscle relaxed, tracheal intubation is performed and mechanical ventilation is settled. Protective ventilation strategy is used. The tidal volume is set as standard body weight × 6–8 mL/kg, and the PEEP is set at 6 cm H_2O. Oxygen is administered at a concentration fraction of < 60%. Every hour and before the end of every procedure, lung recruitment maneuvers are performed.

Anesthesia maintenance

Propofol or etomidate is titrated to maintain Narcotrend index between 24-65 or BIS index between 40–60. Continuous pumping of cisatracurium is initiated 30 min after induction at 1–2 μg/kg/min. Tropisetron is given at the beginning of the procedure.The cisatracurium infusion is discontinued 30 min before the end of surgery (end of the last suture), sufentanil 0.05–0.1 μg/kg is given 15 min before the end of surgery for postoperative analgesia, propofol/etomidate is discontinued 5–10 min before the end of surgery, and remifentanil is discontinued at the end of surgery. Neostigmine antagonism is administered. See Appendix 1 for post-anesthesia extubation guidelines.

Postoperative analgesia

Local incision infiltration with 0.5% ropivacaine and patient-controlled intravenous analgesia (PCIA) are used. The infusion rate of sufentanil for PCA is 0.04 μg/kg/h, the bolus dose is 0.01 μg/kg, and the lockout time is 15 min. If postoperative analgesia is inadequate (VAS ≥ 4), rescue analgesia is given as needed.

3.6 Measurements

Pre-anesthesia

Demographic information: age, sex, height, weight

Medical history: past history, history of anesthesia, ASA classification, comorbidities, diagnoses

Procedure: name of the procedure, type of procedure

Circulatory indicators: baseline BP (especially BP on the higher side at the preoperative visit) and pre-anesthesia vital signs (HR, SBP, diastolic BP [DBP], SpO_2, respiratory rate [RR])

Adrenocortical function hormone levels measured using 5 mL of blood collected before 7:00 am on the day of surgery: cortisol, aldosterone, adrenocorticotropic hormone (ACTH)

Other indicators: preoperative medications, Narcotrend or BIS index, rehydration volume

During anesthesia

Hemodynamic indicators (HR, SBP, DBP): before induction; before the start of the procedure; 1 min after the start of the procedure; after perforation of the pneumoperitoneum; 10 min, 60 min, 120 min, and 180 min after the start of the procedure; and at the end of the procedure

Blood collection at the end of the operation for cortisol, aldosterone, and ACTH (5 mL of blood)

Intraoperative urine output

Record the dosage of etomidate/propofol during induction and maintenance, respectively

Record the amount of rehydration fluid (separated as crystals or colloids) during induction and maintenance, respectively

Other indicators: intraoperative medication combinations and Narcotrend or BIS index

Post-anesthesia

Time to awaken from anesthesia

VAS pain scores and PONV by 6 h postoperatively

Postoperative complications during hospitalization

Satisfaction and comfort with anesthesia determined between 7:00 and 8:00 am on postoperative day 1

Daily record between 7:00 and 8:00 am of PONV and VAS scores for the previous 24 h

Hormone levels: cortisol, aldosterone, and ACTH measured before 7:00 am on the first and third postoperative days (5 mL of blood collected each time)

3.7 Outcomes

***Primary Outcome*：In-hospital major complications after surgery**

All complications that occur postoperatively (during hospitalization) are recorded (see Appendix 3 for diagnostic criteria), and the difference in morbidty between the two groups is compared.

Secondary outcomes

Adrenocortical function: Concentration of COR, ALD and ACTH at 7:00 am on the day of surgery, at the end of surgery, and at 7:00 am on the first and third postoperative days

Quality of recovery from anesthesia: (1) time to response to verbal command, and to extubating the tracheal tube, (2) time to discharge from postanesthesia care unit (PACU) and to discharge from hospital, (3) patients' self-scale of their pain on visual analogue scale, (4) patients' self-scale of their comfort and satisfaction on anesthesia on visual analogue scale (5) patients' score of postoperative nausea and vomiting (PONV)

Hemodynamic stability during anesthesia: proportion of patients with BP fluctuations > ± 20% of the base value

Long-term outcomes: All-cause mortality by 6 months and 12 months after surgery.

3.8 Data Collection

An electronical data capture system is used. After confirming that the established data set is correct, the data will be locked by the principal researcher, the sponsor, the statistical analyst. No further changes can be made to the locked data file. Clinical research coordinators are assigned by the sponsor to monitor the study center at regular intervals to ensure compliance with the study protocol, good clinical practice, and laws and regulations. This includes on-site verification of the completeness and clarity of the CRF, cross-checking with the original records, and clarification of administrative matters.

3.9 Statistical analysis (details in Supplement 2. Statistical analysis plan)

Data set

Full analysis set (FAS) refers to all eligible patients who were randomized to treatment, but does not include drop-out cases. Primary indicators are analyzed according to the intention-to-treat (ITT) principle. Missing values for comparability analyses and secondary efficacy indicators are analyzed according to the actual data obtained.

Per protocol set (PPS) refers to the set of cases that meet the inclusion criteria, do not meet the exclusion criteria, and have completed the treatment protocol. The PPS is a subset of the FAS in which each subject in the

dataset is a valid case or sample with good adherence, no protocol violations (including protocol-violating drugs), and complete baseline values for key indicators.

Safety set (SS) refers to the actual data for subjects who receive at least one treatment after randomization and for whom safety indicators are documented. The incidence of adverse reactions is calculated using the number of cases in the safety set.

Data presentation

Quantitative data: Data will be presented using mean ± standard deviation, median (upper and lower quartiles), and min and max.

Categorical data: Data will be presented using frequencies, composition ratios, or percentages.

Adverse events: Will be analyzed, with the number of cases, category, and severity of occurrences counted separately and their relationship to the drugs in use studied.

3.10 Sample size considerations[16]

According to the results of the preliminary study, the incidence of complications in the etomidate group and propofol group was 7.5% and 8.0%, respectively. The Δ was set as 3% according to the expertise advice. The value of α was 0.025, and value of β was 0.2 (statistical power of 80%). The calculation showed that a total of 917 patients were needed for each group, and the total sample size of this study was 1834. Finally, 1930 patients were required in this study when an ineligible rate of 5% was considered.

Anticipated date of first enrollment: April 2016

Anticipated date of final enrollment: August 2018

4. Appendix

4.1 Appendix 1. Indications for tracheal extubation

A. Normal PaO_2 or SpO_2

B. Normal respiratory pattern. 10-min T-tube ventilation test shows that the patient is able to breathe spontaneously and without effort, with an RR < 30 breaths/min and tidal volume > 300 mL.

C. Consciousness is restored and the airway can be protected

D. Muscle strength is fully restored

4.2 Appendix 2. Postoperative analgesia assessment – VAS score

A scale of 0–10 is used to indicate different levels of pain intensity, with “0” being no pain and “10” being the most severe pain.

Below 4 is mild pain, 4–7 is moderate pain, and above 7 is severe pain.

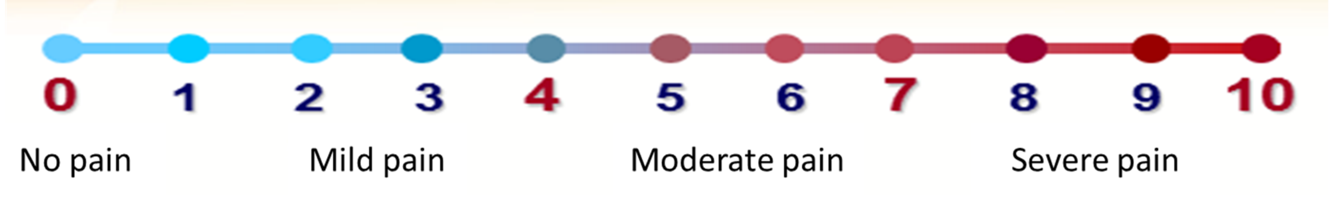

4.3 **Appendix 3. Full list of major complications**[17]**（details in Supplement 3: definitions）**

References

1) DeFrances CJ, Lucas CA, Buie VC, Golosinskiy A. 2006 National Hospital Discharge Survey. *Natl Health Stat Report*. 2008;(5):1–20

2) Singh A, Antognini JF. Perioperative pharmacology in elderly patients. *Curr Opin Anaesthesiol*. 2010;23(4):449–454

3) Turrentine FE, Wang H, Simpson VB, Jones RS. Surgical risk factors, morbidity, and mortality in elderly patients. *J Am Coll Surg*. 2006;203(6):865–877

4) Devereaux PJ, Yang H, Yusuf S, for the POISE Study Group, et al. Effects of extended-release metoprolol succinate in patients undergoing non-cardiac surgery (POISE trial): a randomized controlled trial. *Lancet*. 2008;371(9627):1839–1847

5) McGory ML, Maggard MA, Ko CK. A meta-analysis of perioperative beta blockade: what is the actual risk reduction? *Surgery*. 2005;138(2):171–179

6) Auerbach AD, Goldman L. Beta-blockers and reduction of cardiac events in noncardiac surgery: scientific review. *JAMA*. 2002;287(11):1435–1444

7) Fleischmann KE, Goldman L, Young B, et al. Association between cardiac and noncardiac complications in patients undergoing noncardiac surgery: outcomes and effects on length of stay. *Am J Med*. 2003;115(7):515–520

8) Devereaux PJ, Xavier D, Pogue J, et al. Characteristics and short-term prognosis of perioperative myocardial infarction in patients undergoing noncardiac surgery: a cohort study. *Ann Intern Med*. 2011;154(8):523–528

9) Kulaylat MN, Dayton MT. Surgical complications. In: Townsend CM Jr, Beauchamp RD, Evers BM, Mattox KL (eds) *Sabiston Textbook of Surgery*, 18th edn. 2008. Saunders Elsevier, Philadelphia, PA, pp. 338–347

10) Lindenauer PK, Pekow P, Wang K, et al. Perioperative beta blocker therapy and mortality after major noncardiac surgery. *N Engl J Med*. 2005;353:349–361

11) Skinner DL, Goga S, Rodseth RN, Biccard BM. A meta-analysis of intraoperative factors associated with postoperative cardiac complications. *South Afr J Anaesth Analg*. 2012;18(4):186–191

12) Kheterpal S, O'Reilly M, Englesbe MJ, et al. Preoperative and intraoperative predictors of cardiac adverse events after general, vascular, and urological surgery. *Anesthesiology*. 2009;110(1):58–66

13) Walsh M, Devereaux PJ, Garg AX, et al. Relationship between intraoperative mean arterial pressure and clinical outcomes after noncardiac surgery: toward an empirical definition of hypotension. *Anesthesiology*. 2013;119(3):507–515

14) Mazerolles M, Senard JM, Verwaerde P, et al. Effects of pentobarbital and etomidate on plasma catecholamine levels and spectral analysis of blood pressure and heart rate in dogs. *Fundam Clin Pharmacol*. 1996;10(3):298–303

15) Ebert TJ, Muzi M, Berens R, et al. Sympathetic responses to induction of anesthesia in humans with propofol or etomidate. *Anesthesiology*. 1992;76:725–733

16) Chow S, Shao J, Wang H. *Sample Size Calculations in Clinical Research*, 2nd edn. 2008. Chapman & Hall/CRC Biostatistics Series, p 91

17) Futier E, Lefrant JY, Guinot PG, et al. Effect of Individualized vs Standard Blood Pressure Management Strategies on Postoperative Organ Dysfunction Among High-Risk Patients Undergoing Major Surgery: A Randomized Clinical Trial. *JAMA*. 2017;318(14):1346-1357

Statistical Analysis Plan

Safety and effectiveness of etomidate versus propofol in elderly patients: a multi-center, randomized, controlled study (EPIC study)

Prepared by:
Liyuan Tao, MD, PhD (Research center of clinical epidemiology, Peking university third hospital)
Zhihong Lu, MD, PhD (Department of anesthesiology and perioperative medicine, Xijing hospital)
Hailong Dong, MD, PhD (Department of anesthesiology and perioperative medicine, Xijing hospital)
Lize Xiong, MD, PhD (Department of anesthesiology and perioperative medicine, Xijing hospital)

Table of Contents

1 Introduction
2 Methods
2.1 Funding and Ethical Approval
2.2 Study design
2.3 Randomization and Blinding
3 Study outcome variables
3.1 Primary outcome variables
3.2 Secondary outcome variables
4 Genenral considerations
4.1 Population
4.2 Covariates and subgroup analysis
4.3 Missing data
4.4 Interim analysis and multi-center difference
5 Statistical analysis
5.1 Data management and general analysis
5.2 Analysis of primary endpoint
5.3 Analysis of other endpoints
5.4 Analysis of repeated measurements
5.5 Software and significant level of the statistical analyses

1 Introduction

This multi-center study aims to investigate the effects of etomidate/propofol-based venous anesthesia on intraoperative and postoperative (during hospitalization) complications in elderly patients. The function of adrenal cortex, anesthesia recovery quality, hemodynamic stability during anesthesia, and comfort level and satisfactory level of patients who receive etomidate- versus propofol-based venous anesthesia will also be analyzed.

2 Methods

2.1 Ethical Approval

This study will be conducted by 22 hospitals in China. This study has been registered on the ClinicalTrials website (register number: NCT02910206), and approved by the Ethics Committee of the First Affiliated Hospital of the Fourth Military Medical University (Ethics approval number: KY20162013-2).

2.2 Study design

This is a multi-center, randomized controlled non-inferiority study. The patients will be randomly divided in 1:1 ratio into etomidate group and propofol group. This study aims to investigate whether the intraoperative and postoperative (during hospitalization) incidence of complications in the etomidate group was non-inferior to the propofol group.

2.3 Randomization and Blinding

After signing the informed consent forms, the patients will be enrolled according to the inclusion and exclusion criteria. The patients who meet the eligibility criteria will be randomized into two groups. Local investigators randomly allocated participants after their enrolment using a secure, central web-based randomization system. The random sequence was generated by a computer with a randomized block size of four or six and stratified by center. The allocation concealment was also conducted by the web-based randomization system.

The primary endpoint of this study is the incidence of intraoperative and postoperative (during hospitalization) complications. The patients, the surgeons, the outcome assessors, and the statisticians will be blinded to the group assignment.

3 Study outcome variables

3.1 Primary outcome variables

The overall incidence of intraoperative and postoperative complications will be recorded, and the incidence of major complications pre-defined will be compared between the two groups.

3.2 Secondary outcome variables

Anesthesia recovery quality: 1) time indicators (including time of eye-opening on call, time of extubation, time of post-anesthesia care unit (PACU) admission, time of PACU discharge, time of orientation recovery, and time of hospital discharge); 2) postoperative nausea and vomiting (PONV) score (at 6 h postoperation, and 7:00-8:00 every day after operation). The PONV score is evaluated according to the WHO criteria as follows: 1 point indicates no nausea or vomiting; 2 points indicate mild nausea and abdominal discomfort, but

no vomiting; 3 points indicate evident nausea and vomiting, but with no vomiting contents; and 4 points indicate severe vomiting with contents (such as gastric juice), and requires drug management; and 3) visual analog score (VAS) score (6 h after operation).

Hemodynamic stability during anesthesia: 1) percentage of patients with blood pressure fluctuation ≥20% baseline level; 2) percentage of patients using vasoactive agents; and 3) total volume of intraoperative fluid infusion.

Comfort level and satisfactory level of patients: The patients will be inquired about the comfort level and satisfactory level to anesthesia on day 1 after the operation. The comfort level of patients will be classified from extremely discomfort to very comfort (1-10 points), and the satisfactory level will be classified from extremely unsatisfied to very satisfied (1-10 points).

Function of adrenal cortex: the changes in cortisol, aldosterone, and ACTH at different time points (including before 7:00 on the day of operation, before completion of operation, and before 7:00 on days 1 and 3 after operation). Five centers were selected for collecting the serum sample.

All-cause mortality at Months 6 and 12 after surgery: the all-cause mortality of the patients at 6 and 12 months after the operation was obtained through follow-up.

4 General considerations

4.1 Population

4.1.1 Sample size

The sample size of this study will be calculated according to the method of non-inferiority. The indicators for sample size calculation include the incidence of postoperative (during hospitalization) complications. The equation for calculating the sample size is as follow:

$$n_2 = \frac{(z_{1-\alpha} + z_{1-\beta})^2[p_1(1-p_1)/k + p_2(1-p_2)]}{(p_1 - p_2 + \Delta)^2}$$

In the equation, n1 is the sample size of the etomidate group, n2 is the sample size of the propofol group, p1 is the incidence of complications in the etomidate group, p2 is the incidence of complications in the propofol group, Δ is the cut-off value of non-inferiority, α is the type I error, β is the type II error, and κ is the ratio of the sample sizes of the two groups (n1/n2).

According to the results of a preliminary study, the expected incidence of complications was 7.5% in the etomidate group and 8.0% propofol group. The Δ was set at 3% based on expert advice. Given an α of 0.025 and β of 0.2 (i.e., statistical power of 80%), a total of 917 patients per group (total sample size: 1834 patients) was required to demonstrate non-inferiority. Assuming an ineligibility rate of 5%, 1930 patients were required to be screened to provide an adequate sample size.

4.1.2 Full analysis set and safety set

The major aim of this study is to investigate whether the incidence of postoperative (during hospitalization) complications in the etomidate group is inferior to that of the propofol group. The primary endpoint of this study is the incidence of complications, which is a safety event. Therefore, the full analysis set and safety set in this study is identical.

The full analysis set and the safety set are defined as all patients who are randomized and have intraoperative or postoperative adverse events. The patients who are randomized but did not undergo anesthetic operation will be excluded from the full analysis set and safety set.

The assessment of primary endpoint will be performed based on full analysis set and safety set.

4.1.3 Per protocol set

Patients who receive randomization and underwent consequent operation therapy, receive the anesthetic agents and complete the follow-up according to the study protocol will be included in the per protocol set. The per protocol set is mainly used for sensitivity analysis.

4.2 Covariates and subgroup analysis

As this is an RCT study, the probability of imbalance in baseline data between the two groups is speculated to be 5% (a minor probability event). Therefore, no multivariate analysis with the adjustment of covariates will be performed to analyze primary endpoint. In addition, subgroup analysis will be performed by duration of intervention (anesthesia). The subgroup analysis will be carried out as exploratory.

4.3 Missing data

Regarding the primary endpoint, the intraoperative and postoperative (during hospitalization) complications occur during the operation process or during hospitalization, and thus the probability of missing data associate with primary endpoint remained relatively low. In addition, all-cause mortality at Months 6 and 12 after surgery were also collected through follow-up. There may be missing data.

If missing data were found, the percentage of missing data will be reported, the potential patterns of missing data should be examined, and appropriate method should be used for imputation of missing data. The multiple imputation method will be preferred for analyzing the missing data, and the results should be reported in the manuscript as sensitivity analysis. The patients' demographic characteristics will be involved in the imputed missing data model for multiple imputation, and the number of multiple imputations will be set as 5.

4.4 Interim analysis

No interim analysis is planned in this study.

4.5 multi-center effect analysis

This is a multicenter RCT study, and there may be center effects among different centers. The Generalized Linear Mixed Model (GLMM) will be used to control the multi-center effect, with the different centers set as fixed effect.

5 Statistical analysis

5.1 Data management and general analysis

Electronic dataset system will be used for data collection and management. Independent data management committee and data monitoring board is responsible for the management of validity and effectiveness of the data.

The data analyses mainly include statistical description and statistical inference. Quantitative data will be described by central

tendency and dispersion tendency. The normally distributed data of central tendency and dispersion tendency will be described as means and standard division, respectively. The non-normally distributed data of central tendency and dispersion tendency will be described as median and quartiles. The qualitative data will be described as frequency and percentage. Statistical inference, independent t test or non-parameter test will be used to compare the quantitative data between the two groups, while chi-square test or Fisher's exact test will be used for comparing the qualitative data between the two groups.

5.2 Analysis of primary endpoint

For primary endpoint comparison, the SS analysis will be performed to evaluate the differences between the two groups on major complications during hospital stay. The absolute rate difference (RD) with 95% CI will be estimated for the primary outcomes. The 95% CIs of absolute RDs will be calculated using the Newcombe-Wilson score method. The per-protocol (PP) sets will be also used for sensitivity analysis.

5.3 Analysis of other endpoints

For secondary and exploratory endpoints, continuous data will be presented as means (SDs) or median (IQRs), as appropriate. The secondary endpoint followed no Gaussian distribution, will be presented as median (interquartile range) and tested by Mann-Whitney U test. The mean (95%CIs) of between-group differences of the median will be calculated by bootstrap method (1000 replications). For all-cause mortality, the Kaplan-Meier survival curve and log-rank test will be used to detect the difference between groups.

5.4 Analysis of repeated measurements

Regarding adrenal hormone levels, 300 patients will be randomly selected, and the blood samples will be obtained at four time points, which were used to measure the levels of hormones, including serum cortisol (COR), aldosterone (ALD), and adreno-cortico-tropic-hormone (ACTH). These data will be analyzed by analysis of variance (ANOVA) for repeated measurements. In addition, the differences between the two groups at different time points will also be analyzed.

5.5 Software and significant level of the statistical analyses

All statistical analyses will be conducted using SPSS 18.0 (IBM, Armonk, NY, USA) and R 3.4.0 software (The R Project for Statistical Computing, www.r-project.org). Besides, the tests will be corrected using Bonferroni correction. Statistical significance is set as $P<0.05$ with 2-sided testing.

Regarding the comparison of blood pressure between the two groups, the significant level will be adjusted by Bonferroni correction, as the blood pressure will be measured many times in this study. The significant level after correction will be calculated by $\alpha' = \alpha/K$, in which $\alpha=0.05$, and K is the number of times of comparisons.
