# Supplementary material for: Effect of Etomidate vs Propofol for Total Intravenous Anesthesia on Major Postoperative Complications in Older Patients: A Randomized Clinical Trial
Source: JAMA Surg. 2022 Aug 10;157(10):888–95. doi: 10.1001/jamasurg.2022.3338 (PMC9366659; doi:10.1001/jamasurg.2022.3338)
Supplement: Supplement 2. — eMethods. Definition of Major Complications eReference eTable 1. Subgroup Analysis of the Effect of Etomidate vs Propofol on Major In-Hospital Complications (Safety Set) eTable 2. Mean (SD) Pain, Patient Satisfaction with Anesthesia and Patient Comfort Scores (Safety Set) eTable 3. ANOVA for Repeated Measurements of Serum Cortisol, Aldosterone and ACTH (Safety Set) eTable 4. Primary and Secondary End Points (Per Protocol Set) eTable 5. Effect of Etomidate vs Propofol on Individual Complications (Safety Set) eFigure 1. Blood Pressure, Heart Rate and Pulse Oximetry During Anesthesia (Safety Set) eFigure 2. Kaplan-Meier Curves of Survival After Surgery (Safety Set) [file jamasurg-e223338-s002.pdf]

Supplementary Online Content

Lu Z, Zheng H, Chen Z, et al. Effect of etomidate vs propofol for total intravenous anesthesia on major postoperative complications in older patients: a randomized clinical trial. *JAMA Surg*. Published online August 10, 2022. doi:10.1001/jamasurg.2022.3338

eMethods. Definition of Major Complications

eReference

eTable 1. Subgroup Analysis of the Effect of Etomidate vs Propofol on Major In-Hospital Complications (Safety Set)

eTable 2. Mean (SD) Pain, Patient Satisfaction with Anesthesia and Patient Comfort Scores (Safety Set)

eTable 3. ANOVA for Repeated Measurements of Serum Cortisol, Aldosterone and ACTH (Safety Set)

eTable 4. Primary and Secondary End Points (Per Protocol Set)

eTable 5. Effect of Etomidate vs Propofol on Individual Complications (Safety Set)

eFigure 1. Blood Pressure, Heart Rate and Pulse Oximetry During Anesthesia (Safety Set)

eFigure 2. Kaplan-Meier Curves of Survival After Surgery (Safety Set)

This supplementary material has been provided by the authors to give readers additional information about their work.

© 2022 Lu Z et al. *JAMA Surgery.*

eMethods. Definition of Major Complications

The primary outcome measure was a composite of major postoperative complications in hospital, defined as one or more of the following:[1]

Cardiovascular complications	Severe arrhythmia, acute heart failure or myocardial infarction
Pulmonary complications	Hypoxemia, need for non-invasive or invasive mechanical ventilation, ARDS, pneumonia
Neurologic complications	Stroke or altered consciousness
Gastrointestinal complications	Liver failure, gastrointestinal bleeding or perforation
Urological complications	Acute kidney dysfunction
Hematological complications	SOFA sub-score of 2 points or more in the coagulation component (platelets <100 × 10^3/mm^3)
Thromboembolic complications	Deep venous thrombosis, pulmonary embolism
Infectious complications	Anastomotic leak, surgical site infection, urinary tract infection, sepsis, severe sepsis and septic shock
Death	All-cause death

1. **Cardiovascular complication (severe arrhythmia, acute heart failure or myocardial infarction)**

Heart failure: signs and symptoms of left or right heart failure, effective treatment, or cardiac ultrasound findings of left ventricular ejection fraction less than 35%, suggesting left ventricular impairment.

Acute infarction: myocardial enzyme profile suggestive of at least one index (commonly cardiac troponin [cTn]) more than 5 times the 99% recommended upper limit and meeting one of the following indicators: ischemic symptoms; ECG changes indicating new ischemia (new ST-T changes or left bundle branch block [LBBB]); cardiac imaging suggestive of new myocardial loss or new ventricular wall motion abnormalities; coronary angiography; or autopsy suggestive of intracoronary thrombus.

Late infarction: ST-segment elevation or T-wave changes; pathological Q waves on more than two ECG leads; new LBBB on ECG; cTn exceeding the recommended upper limit.

Severe arrhythmias: ventricular tachycardia, supraventricular tachycardia, atrial fibrillation, etc., diagnosed by electrocardiogram.

Re-vascularization: including PCI, CPB-CABG, OP-CABG.

2. **Pulmonary complications (hypoxemia, need for non-invasive or invasive mechanical ventilation, ARDS, pneumonia)**

Hypoxemia was defined as a PaO_2 <60 mmHg or SpO_2 <90% on room air.

Noninvasive ventilation: Noninvasive ventilation will be considered in case of presence and persistence for more than 30 minutes of hypoxemia (as defined above) and at least one of the following:

a) Respiratory rate higher than 30/min

b) Clinical signs suggestive of intense respiratory muscle work and/or labored breathing, such as use of accessory respiratory muscles, paradoxical motion of the abdomen, or intercostal retraction.

Pneumonia: Pneumonia was suspected upon the presence of new and/or progressive pulmonary infiltrates on chest radiograph plus two or more of the following criteria:

a) Fever ⩾38.5° C or hypothermia <36°C

b) Leukocytosis ⩾12000 WBC/mm^3 or leukopenia <4000 WBC/mm^3

3. **Neurologic complications (stroke or altered consciousness)**

© 2022 Lu Z et al. *JAMA Surgery.*

Postoperative altered consciousness was determined clinically by the treating physician, and defined as a Glasgow Coma Scale (GCS) score of 14 or less (SOFA sub-score of 1 point or more in the neurologic component). Additionally, whenever possible, the Digital Symbol Substitution Test (DSST) score was recorded.

Acute ischemic stroke was defined as an acute new focal neurologic deficit with confirmation by CT scan and/or MRI.

4. **Gastrointestinal complications (liver failure, gastrointestinal bleeding or perforation)**

Liver failure: total plasma bilirubin >180 mmol/L, glutathione and glutamic oxalacetic transaminase more than 2 times the 99% recommended upper limit, reduced plasma cholinesterase, elevated blood ammonia levels, hepatic encephalopathy.

Gastrointestinal bleeding: gastrointestinal tract bleeding requiring a blood transfusion.

Gastrointestinal perforation: perforation of a cavernous organ requiring surgical treatment.

5. **Urological complications (acute kidney dysfunction: RIFLE stage risk or higher)**

RIFLE classification

**Class**	**Glomerular Filtration Rate criteria**	**Urine output criteria**
Risk	Serum creatinine increase × 1.5	<0.5 ml/kg/h × 6h
Injury	Serum creatinine increase × 2	<0.5 ml/kg/h × 12h
Failure	Serum creatinine increase × 3, or serum creatinine ⩾4 mg/dL (with an acute rise >0.5 mg/dL)	<0.3 ml/kg/h × 24h, or anuria × 12h
Loss	Persistent acute renal failure = complete loss of kidney function	
End-stage kidney disease	End-stage kidney disease >3 months	

© 2022 Lu Z et al. *JAMA Surgery.*

6. **Hematological complications (SOFA sub-score of 2 points or more in the coagulation component (platelets <100 × $10^3/mm^3$)**

Sequential Organ Failure Assessment (SOFA) score

**Organ system**	**Score**				
	**0**	**1**	**2**	**3**	**4**
Respiration					
PaO_2/FiO_2, mm Hg	≥400	<400	<300	<200	<100 with respiratory support
Coagulation					
Platelets, × $10^3/mm^3$	≥150	<150	<100	<50	<20
Liver					
Bilirubin μmol/L	<20	20–32	33–101	102–204	>204
Cardiovascular					
Hypotension	MAP ≥70 mm Hg	MAP <70 mm Hg	Dopamine ≤5.0 (μg/kg/min) or any dose dobutamine	Dopamine >5.0 (μg/kg/min) or noradrenaline ≤0.1 or adrenaline ≤ 0.1	Dopamine >15.0 (μg/kg/min) or adrenaline >0.1 or noradrenaline >0.1
Central nervous system					
Glasgow Coma Scale score	15	13-14	10-12	6-9	<6
Renal					
Creatinine, μmol/L urine output	<110	110–170	171–299	300–440<br><500 mL/day	>440<br><200 mL/day

7. **Thromboembolic complications (deep venous thrombosis, pulmonary embolism)**
8. **Infectious complications (surgical site infection, urinary tract infection, sepsis, severe sepsis and septic shock)**

Sepsis was defined as: a) defined focus of infection and b) at least two systemic inflammatory response syndrome (SIRS) criteria. Defined focus of infection was indicated by either an organism grown in blood or sterile site, or an abscess or infected tissue (e.g. pneumonia, peritonitis, urinary tract, vascular line infection, soft tissue, etc.).

Severe sepsis was defined by sepsis plus at least one organ failure, hypotension or hypoperfusion. Septic shock was sepsis-induced hypotension despite adequate fluid resuscitation along with the presence of perfusion abnormalities.

Criteria for surgical site infection (SSI): surgical infection site within 30 days after the operative procedure was defined according to the criteria of the Centers for Disease Control and Prevention (CDC).

9. **Death (all-cause death)**

eReference

1. Futier E, Lefrant JY, Guinot PG, et al. Effect of individualized vs standard blood pressure management strategies on postoperative organ dysfunction among high-risk patients undergoing major surgery: A randomized clinical trial. *JAMA*. 2017;318(14):1346-1357.

© 2022 Lu Z et al. *JAMA Surgery.*

eTable 1. Subgroup Analysis of the Effect of Etomidate vs Propofol on Major In-Hospital Complications (Safety Set)

Subgroups	In-Hospital Major Complications, No. (%)		*P* Value	Rate Difference (95% CI), %
	Etomidate (n = 967)	Propofol (n = 950)		
Duration of surgery				
≤2 hours	34/492 (6.9)	35/479 (7.3)	.81	-0.4 (-3.1 to 2.3)
>2 hours	55/475 (11.6)	48/471 (10.2)	.49	1.4 (-1.9 to 4.7)
Duration of anesthesia				
≤2 hours	28/413 (6.8)	29/390 (7.4)	.72	-0.7 (-3.6 to 2.3)
>2 hours	62/554 (11.2)	54/560 (9.6)	.40	1.5 (-1.5 to 4.6)
ASA status				
I or II	63/742 (8.5)	56/705 (7.9)	.71	0.5 (-1.8 to 2.9)
III	27/225 (12.0)	27/245 (11.0)	.74	1.0 (-3.9 to 5.8)

Abbreviations: ASA, American Society of Anesthesiologists; CI, confidence interval.

© 2022 Lu Z et al. *JAMA Surgery.*

eTable 2. Mean (SD) Pain, Patient Satisfaction with Anesthesia and Patient Comfort Scores (Safety Set)

**Outcome**	**Etomidate (n = 967)**	**Propofol (n = 950)**	***P* Value**	**Difference (95% CI)**
**Pain**				
Hour 6 post-op	2.4 (1.4)	2.3 (1.4)	.07	0.1 (-0.1 to 0.2)
Day 1 post-op	2.1 (1.3)	2.0 (1.2)	.53	-0.0 (-0.1 to 0.1)
Day 3 post-op	1.1 (1.1)	1.1 (1.2)	.34	-0.1 (-0.2 to 0.1)
**Patient satisfaction with anesthesia**				
Day 1 post-op	8.1 (1.2)	8.1 (1.2)	.29	0.1 (-0.1 to 0.2)
**Patient comfort**				
Day 1 post-op	8.0 (1.3)	7.9 (1.3)	.03	0.1 (0.0 to 0.2)

Abbreviations: SD, standard deviation; CI, confidence interval;

© 2022 Lu Z et al. *JAMA Surgery.*

eTable 3. ANOVA for Repeated Measurements of Serum Cortisol, Aldosterone and ACTH (Safety Set)

	**Etomidate (n = 967)**		**Propofol (n = 950)**		**P_b value**
Cortisol (μg/dL)		P_a value		P_a value	0.17
Day of surgery	7.1 ± 3.1	ref.	6.8 ± 3.1	ref.	
End of anesthesia	4.8 ± 2.7	<0.001	6.1 ± 3.4	0.04	
Day 1 post-op	6.8 ± 4.6	0.62	8.0 ± 5.1	0.007	
Day 3 post-op	7.9 ± 5.1	0.03	7.6 ± 4.3	0.009	
Aldosterone (ng/dL)					0.07
Day of surgery	0.15 ± 0.05	ref.	0.15 ± 0.10	ref.	
End of anesthesia	0.13 ± 0.05	<0.001	0.15 ± 0.07	0.29	
Day 1 post-op	0.14 ± 0.04	0.42	0.16 ± 0.06	0.02	
Day 3 post-op	0.14 ± 0.05	0.43	0.16 ± 0.07	0.17	
ACTH (pg/mL)					0.87
Day of surgery	22.1 ± 25.8	ref.	25.3 ± 31.6	ref.	
End of anesthesia	42.1 ± 94.1	0.008	36.3 ± 79.0	0.07	
Day 1 post-op	57.9 ± 115.0	0.002	31.8 ± 72.9	0.29	
Day 3 post-op	23.8 ± 50.0	0.86	31.6 ± 58.4	0.33	

Abbreviations: Abbreviations: SEM, standard error of mean; ACTH, adrenocorticotropic hormone; P_a, comparison with baseline; P_b comparison between the two groups; ref., reference.

© 2022 Lu Z et al. *JAMA Surgery.*

eTable 4. Primary and Secondary End Points (Per Protocol Set)[a]

**Outcomes**	**Etomidate** (n = 951)	**Propofol** (n = 938)	***P*** **Value**	**Difference (95% CI), %**
**Major in-hospital complications**				
Composite (primary endpoint)	89 (9.4)	83 (8.8)	.70	0.5 (-1.7 to 2.7)
Cardiovascular	1 (0.1)	1 (0.1)	>.99	0.0 (-0.3 to 0.2)
Pulmonary	19 (2.0)	5 (0.5)	<.001	1.5 (0.6 to 2.3)
Gastrointestinal	31 (3.3)	45 (4.8)	.09	-1.5 (-3.0 to -0.1)
Neurological	9 (0.9)	5 (0.5)	.30	0.4 (-0.2 to 1.1)
Urological	1 (0.1)	0 (0.0)	>.99	0.1 (-0.1 to 0.3)
Infectious	19 (2.0)	12 (1.3)	.22	0.7 (-0.2 to 1.7)
Hematological	19 (2.0)	19 (2.0)	.97	0.0 (-1.1 to 1.0)
Thrombosis	2 (0.2)	1 (0.1)	>.99	0.1 (-0.2 to 0.4)
Death	0 (0.0)	0 (0.0)	–	–
**During surgery**				
Hypertension	98 (10.3)	33 (3.5)	<.001	6.8 (4.9 to 8.7)
Hypotension	23 (2.4)	100 (10.7)	<.001	-8.2 (-10.1 to-6.4)
**During emergence**				
Time to response to verbal command, minutes	10.9 (4.4 to 21.4)	12.0 (5.0 to 23.5)	.14	-0.6 (-1.6 to 0.2)
Duration of PACU stay, minutes	36.5 (20.0 to 53.5)	37.3 (22.0 to 55.5)	.15	-1.7 (-4.0 to 0.5)
**During recovery**				
PONV ⩾3				
6 hours post-op	130 (13.7)	131 (14.0)	.90	-0.3 (-2.9 to 2.3)
1 day post-op	61 (6.4)	64 (6.8)	.74	-0.4 (-2.3 to 1.5)
3 days post-op	7 (0.7)	12 (1.3)	.24	-0.5 (-1.3 to 0.2)
Pain				
6 hours post-op	2 (2 to 3)	2 (1 to 3)	.06	0.0 (0.0 to 0.0)
1 day post-op	2 (1 to 3)	2 (1 to 3)	.78	0.0 (0.0 to 0.0)
3 days post-op	1 (0 to 2)	1 (0 to 2)	.39	0.0 (0.0 to 0.0)
Patient satisfaction with anesthesia				
1 day post-op	8 (8 to 9)	8 (7 to 9)	.24	0.0 (0.0 to 0.0)
Patient comfort				
1 day post-op	8 (7 to 9)	8 (7 to 9)	.01	0.0 (0.0 to 0.0)
**Mortality**[b]				
Month 6 post-op	15 (2.3)	20 (3.0)	.38	-0.8 (-2.3 to 0.7)
Month 12 post-op	22 (3.3)	26 (4.0)	.54	-0.7 (-2.4 to 1.1)

[a] Categorical variables are presented as No. (%) and continuous variables as median (IQR).

[b] Long-term survival data were available for 661 patients in the etomidate group and 657 patients in the propofol group.

Abbreviations: CI, confidence interval; PACU, post-anesthesia care unit; PONV, postoperative nausea and vomiting; post-op, post-operation.

© 2022 Lu Z et al. *JAMA Surgery.*

eTable 5. Effect of Etomidate vs Propofol on Individual Complications (Safety Set)

**Outcome, No. (%)**	**Etomidate**<br>**(n = 967)**	**Propofol**<br>**(n = 950)**	***P* Value**	**Risk Difference (95% CI), %**
Heart failure	1 (0.1)	0 (0)	.32	0.1 (-0.3 to 0.6)
Myocardial infarction	0 (0.0)	1 (0.1)	.31	-0.1 (-0.6 to 0.3)
Pneumonia	19 (2.0)	3 (0.3)	.001	1.7 (0.7 to 2.8)
Hypoxia	0 (0.0)	2 (0.2)	.15	-0.2 (-0.8 to 0.2)
Liver failure	28 (2.9)	43 (4.5)	.06	-1.6 (-3.4 to 0.7)
Gastrointestinal bleeding	3 (0.3)	1 (0.1)	.33	0.2 (-0.3 to 0.8)
Gastrointestinal perforation	1 (0.1)	1 (0.1)	.99	0.0 (-0.5 to 0.5)
Delirium	7 (0.7)	4 (0.4)	.38	0.3 (-0.5 to 1.0)
Drowsiness	2 (0.2)	1 (0.1)	.57	0.1 (-0.4 to 0.7)
Acute kidney dysfunction	1 (0.1)	0 (0.0)	.32	0.1 (-0.3 to 0.6)
Sepsis	16 (1.7)	9 (0.9)	.21	0.7 (-0.4 to 1.8)
Severe sepsis	2 (0.2)	2 (0.2)	.99	0.0 (-0.6 to 0.6)
Anastomotic leak	0 (0.0)	1 (0.1)	.31	-0.1 (-0.6 to 0.3)
Surgical site infection	1 (0.1)	0 (0.0)	.32	0.1 (-0.3 to 0.6)
Low platelets	19 (2.0)	19 (2.0)	.96	-0.04 (-1 to 1)
Pulmonary embolism	2 (0.2)	0 (0.0)	.16	0.2 (-0.2 to 0.8)
Deep venous thrombosis	0 (0.0)	1 (0.1)	.31	-0.1 (-0.6 to 0.3)

Abbreviations: CI, confidence interval

© 2022 Lu Z et al. *JAMA Surgery.*

eFigure 1. Blood Pressure, Heart Rate and Pulse Oximetry During Anesthesia (Safety Set)

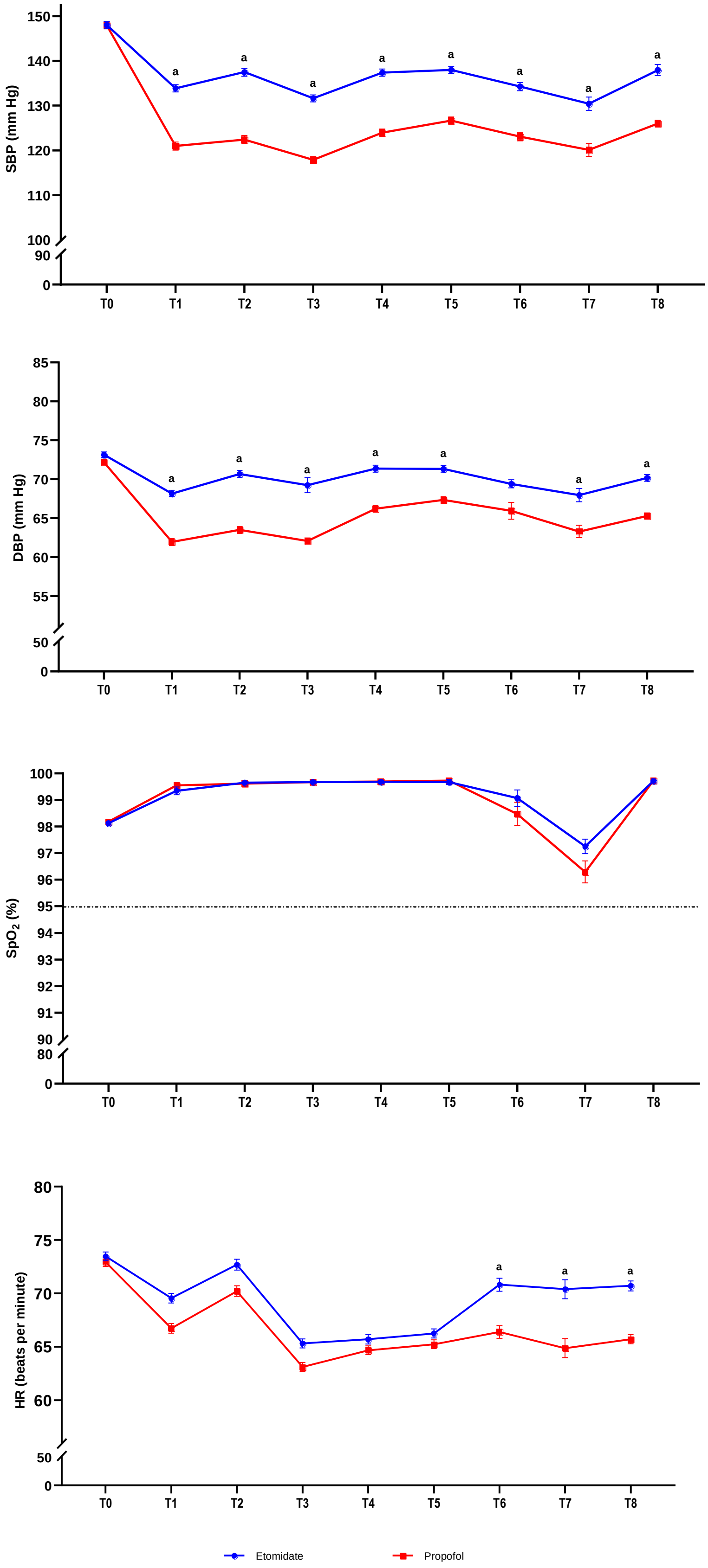

Abbreviations: SEM, standard error of mean; DBP, diastolic blood pressure; HR, heart rate; SBP, systolic blood pressure; SpO_2, oxygen saturation; T0, baseline; T1, before intubation; T2, after intubation; T3, before incision; T4, 1 minute after incision; T5, 60 minutes after incision; T6, 120 minutes after incision; T7, 180 minutes after incision; T8, end of surgery.

[a] Significant difference between the two groups.

© 2022 Lu Z et al. *JAMA Surgery.*

eFigure 2. Kaplan-Meier Curves of Survival After Surgery (Safety Set)

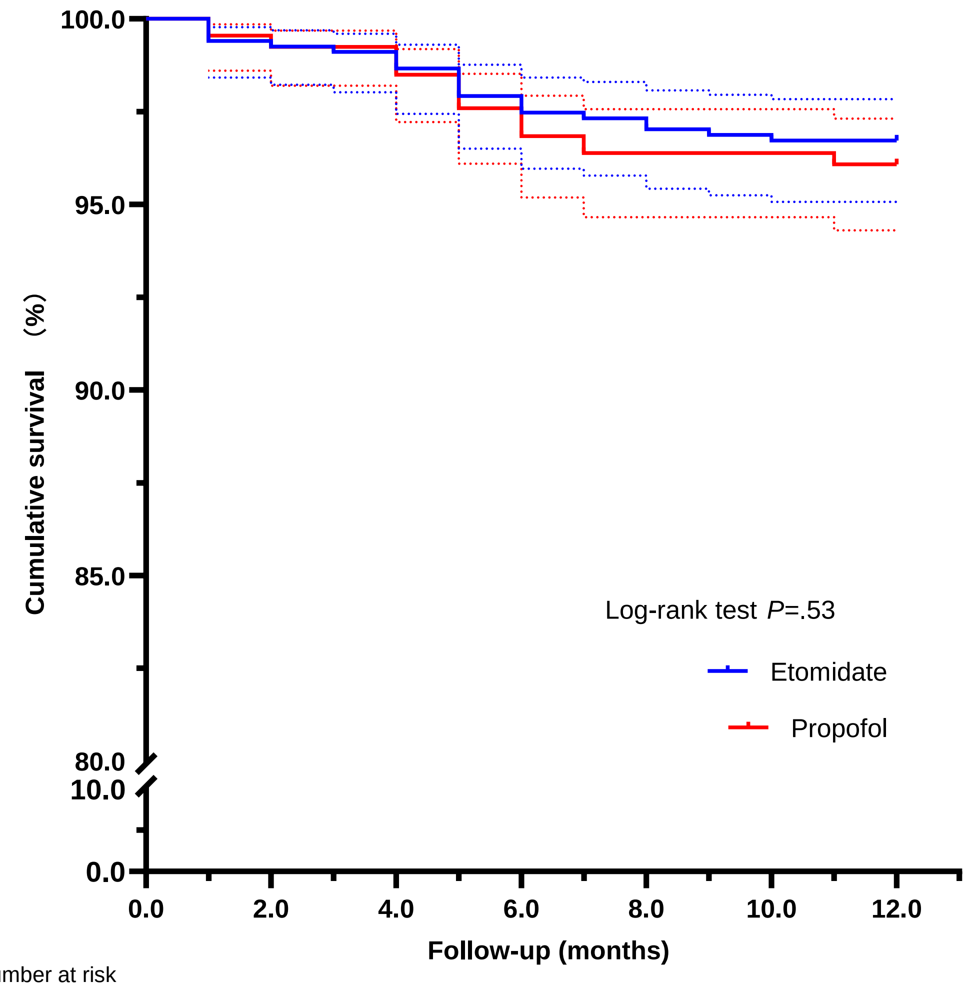

Number at risk

Etomidate	672	668	667	666	663	658	655	654	652	651	650	650	650
Propofol	664	661	659	659	654	648	643	640	640	640	640	638	638

Dotted lines depict the 95% CI of the Kaplan-Meier estimate.

© 2022 Lu Z et al. *JAMA Surgery.*
