# Supplementary material for: Effect of Etomidate vs Propofol for Total Intravenous Anesthesia on Major Postoperative Complications in Older Patients: A Randomized Clinical Trial
Source: JAMA Surg. 2022 Aug 10;157(10):888–95. doi: 10.1001/jamasurg.2022.3338 (PMC9366659; doi:10.1001/jamasurg.2022.3338)
Supplement: Supplement 3. — Data Sharing Statement [file jamasurg-e223338-s003.pdf]

# Data Sharing Statement

Lu. Effect of Etomidate vs Propofol for Total Intravenous Anesthesia on Major Postoperative Complications in Older Patients. *JAMA Surg*. Published August 10, 2022.
doi:10.1001/jamasurg.2022.3338

**Data**
**Data available:** Yes
**Data types:** Deidentified participant data
**How to access data:** How to access data: mzkxlz@126.com; hldong6@hotmail.com
**When available:** With publication

**Supporting Documents**
**Document types:** None

**Additional Information**
**Who can access the data:** Who can access the data: Anyone requesting
**Types of analyses:** Types of analyses: For verification
**Mechanisms of data availability:** Mechanisms of data availability: With signed data access agreement and approval of a proposal
**Any additional restrictions:** None
